# A spatiotemporal single‐cell atlas of porcine development reveals regulatory dynamics and cellular targets of domestication

**DOI:** 10.1002/imt2.70135

**Published:** 2026-06-04

**Authors:** Rong Zhou, Zishuai Wang, Chenghao Hu, Shuhan Deng, Changyun Cai, Yanfang Wang, Shang‐Tong Li, Lijing Bai, Kui Li

**Affiliations:** ^1^ The State Key Laboratory of Animal Biotech Breeding, Institute of Animal Science Chinese Academy of Agricultural Sciences Beijing China; ^2^ College of Agriculture and Biology Liaocheng University Liaocheng China; ^3^ Shenzhen Branch, Guangdong Laboratory for Lingnan Modern Agriculture, Genomics Institute at Shenzhen Chinese Academy of Agricultural Sciences Shenzhen China; ^4^ Glbizzia Biosciences Co. Ltd Beijing China; ^5^ MOA Key Laboratory of Animal Virology, Center for Veterinary Sciences, College of Animal Sciences Zhejiang University Hangzhou China

**Keywords:** cross‐species comparison, developmental atlas, domestication selection, immune trajectory, pig, single‐cell transcriptomics

## Abstract

Systematic characterization of cellular gene expression in livestock tissues during development is essential for understanding the regulation of complex traits. Despite the comprehensive profiling of cell atlases in livestock, a dynamic view of tissue development remains lacking. Here, using both single‐cell and single‐nucleus RNA sequencing, we present a comprehensive single‐cell transcriptomic landscape of 252,033 cells/nuclei, mapping 83 distinct cell types across five pig tissues from prenatal to postnatal developmental stages. Our findings highlight the coordinated remodeling of tissue architecture through stem/progenitor cell proliferation, lineage specification, and functional maturation during organogenesis. We identified key transcription factors and regulatory networks that drive lineage‐specific and spatiotemporally dynamic transcriptional programs. Developmental trajectory analysis identified a conserved bifurcated transcriptional organization of immune cells, accompanied by dynamic changes in transcription factors associated with immune cell maturation. Integrative analysis utilizing multi‐omic, single‐cell, and pig population genomics data identified a muscle‐specific enhancer of the *MYOT* gene as a target of artificial selection, underlying meat‐quality divergence between Asian and European pig breeds. Moreover, cross‐species comparison between pigs and humans revealed conserved cell types, underscoring the evolutionary link. In summary, the comprehensive pig developmental cell atlas serves as a key resource for understanding livestock development, provides insights for precision breeding, and highlights the value of the reference pig cell atlas as a powerful resource for biomedical research.

## Highlights

- A dynamic single‐cell atlas of pig development maps 83 cell types across five tissues and developmental stages.
- Key transcription factors driving lineage specification and functional tissue maturation are identified, and a conserved bifurcated transcriptional trajectory of immune cells was reconstructed.
- Integrative multi‐omic and single‐cell data analysis identifies a muscle‐specific enhancer of the *MYOT* gene as a target of artificial selection that underlies meat quality divergence between Asian and European pig breeds.
- Cross‐species comparison between pig and human reveals conserved cell types and underscores the value of the reference pig cell atlas as a resource for biomedical research.

## Graphical abstract

This study presents a cross‐tissue, single‐cell/single‐nucleus transcriptomic atlas spanning from prenatal to postnatal developmental stages in pig, characterizing 83 distinct cell types from 252,033 cells. Through the construction of cross‐tissue transcriptional regulatory networks and pseudotime trajectories, we revealed that organogenesis is driven by the coordinated processes of stem/progenitor cell proliferation, lineage specification, and functional maturation. Trajectory analysis further uncovered a conserved transcriptional bifurcation pattern associated with immune cell diversification. By integrating population genomics data, a muscle‐specific enhancer near the *MYOT* gene was identified that shows signatures of selection and is associated with divergence in meat quality. Cross‐species comparisons confirmed substantial cellular conservation with humans. Collectively, this atlas serves as a key resource for understanding mammalian development, informing precision breeding strategies, and advancing the pig as a biomedical model.

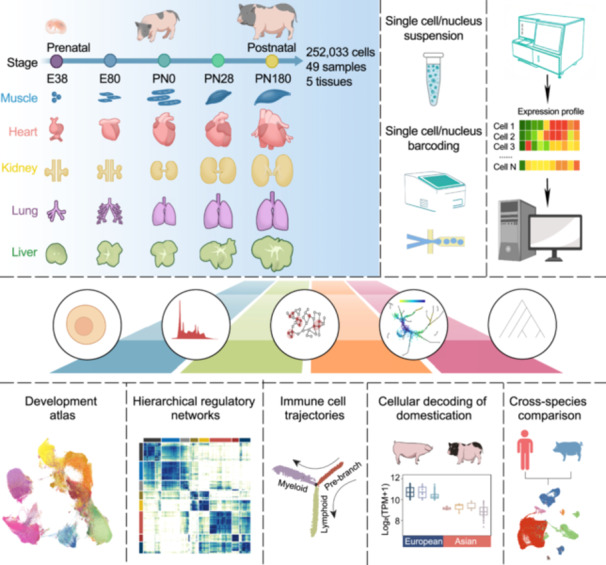

## INTRODUCTION

The pig (*Sus scrofa*) is both an economically important livestock species and a valuable biomedical model due to its close anatomical, physiological, and immunological resemblance to humans [1, 2, 3, 4]. Understanding the genetic regulation of porcine development is therefore critical for advancing both agricultural improvement and human disease modeling [5, 6, 7]. Despite this importance, our knowledge of pig developmental biology at the cellular and molecular levels remains fragmented.

Recent advances in single‐cell RNA sequencing (scRNA‐seq) and single‐nucleus RNA sequencing (snRNA‐seq) have provided new insights into cellular complexity and tissue heterogeneity in pigs [8, 9]. For instance, scRNA‐seq of multiple porcine tissues, including liver [10, 11], lung [12, 13], skin [14], kidney [15, 16], and skeletal muscle [17, 18], has revealed broad cellular heterogeneity and uncovered the transcriptional signatures of diverse cell populations. Similarly, multi‐tissue snRNA‐seq has revealed shared and tissue‐specific regulatory programs underlying organ function [9, 19, 20, 21]. These studies have provided important insights into organ function and cell‐state diversity, yet these efforts have largely focused on individual tissues or specific stages. As a result, the coordinated cellular and molecular dynamics that shape multiple organs throughout pig development remain insufficiently resolved.

Development is a dynamic and tightly regulated process involving complex temporal and spatial coordination of gene expression across multiple tissues [22, 23, 24, 25]. Single‐cell developmental atlases have illuminated stage‐specific changes in cellular composition [26, 27] and transcriptional regulation in several species [28, 29, 30, 31, 32, 33], revealing key molecular events such as endothelial lineage specification [21]. Recent single‐cell studies of individual tissues have provided valuable insights into these processes—for example, identifying cell‐specific transcription factors (TFs) involved in liver development [10] and oogenesis [28]. Yet, a comprehensive cross‐tissue framework capturing these dynamics in pigs is still lacking.

Here, we present a high‐resolution single‐cell and single‐nucleus transcriptomic atlas of porcine development, encompassing five representative organs/tissues, including liver, lung, kidney, heart, and skeletal muscle, across five developmental stages from embryogenesis to adulthood. This dataset, comprising over 250,000 high‐quality transcriptomes, maps 83 cell types and enables an integrative view of cellular composition, lineage trajectories, and regulatory landscapes during pig development. By applying high‐dimensional weighted gene co‐expression network analysis and transcriptional regulon inference, we identified modular gene programs and key TFs associated with lineage specification and functional maturation, and reconstructed a conserved bifurcated transcriptional trajectory of immune cells. Furthermore, coupling our single‐cell atlas with population‐genomic data identified a muscle‐specific enhancer of the *MYOT* gene as a target of artificial selection underlying meat‐quality divergence between Asian and European pig breeds. Cross‐species comparison between pig and human further revealed conserved cell types and underscored the value of the reference pig cell atlas as a resource for biomedical research. This integrative resource offers a foundational reference for dissecting developmental mechanisms, improving breeding strategies, and advancing the use of pigs as translational models in biomedical research.

## RESULTS

### A single‐cell atlas of pig development reveals spatiotemporal dynamics of cellular composition

**Figure 1:**
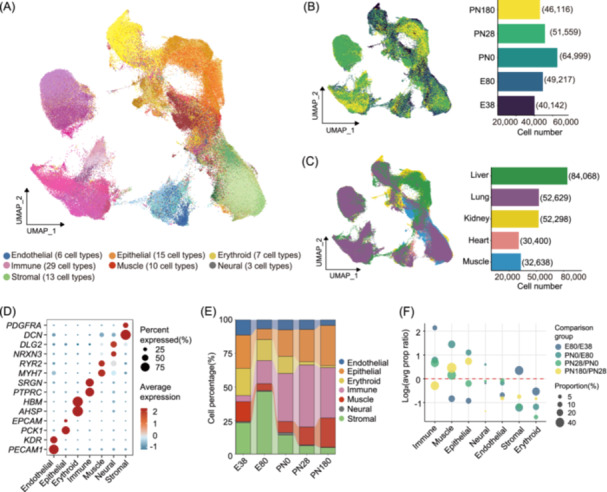
Construction of a spatiotemporal cell atlas during pig development. (A) Uniform manifold approximation and projection (UMAP) visualization of all cell clusters colored by seven major cell lineages. The counts of cell types per lineage are listed. Global clustering of all cells from the dataset, visualized by UMAP, colored by developmental stage (B) or tissue origin (C) (left), with bar plots showing the number of high‐quality cells/nuclei profiled at each group (right). (D) Dot plot of lineage‐specific expressed marker genes. The dot size represents the fraction of cells expressing the gene. The color indicates mean average expression. (E) Proportional distribution of cell lineages across developmental stages. (F) Change of the average ratio of cell type percentage between adjacent time points across five tissues. The color of each point indicates the pair of adjacent time points being compared, while the size of the point reflects the mean cell percentage of the compared time points. Embryonic day 38 (E38), embryonic Day 80 (E80), postnatal Day 0 (PN0), postnatal Day 28 (PN28), postnatal Day 180 (PN180).

To characterize the cellular landscape of pig development, we generated a comprehensive single‐cell and single‐nucleus transcriptomic atlas of pig development across five organs/tissues (liver, lung, kidney, heart, and longissimus dorsi muscle (LDM)) and five developmental stages (embryonic Day 38 and 80, postnatal Day 0, 28, and 180). Following stringent quality control (Methods), 252,033 high‐quality single cells/nuclei transcriptomes were obtained (Tables S1 and S2). The uniform manifold approximation and projection (UMAP) revealed that cells were segregated by tissue identity and developmental stage, indicating substantial spatiotemporal influences on the transcriptome (Figure 1A–C). Based on the expression of canonical marker genes (Tables S3 and S4), we annotated 83 distinct cell types based on the cell type‐specific expression of 431 canonical marker genes, and the 83 cell types were categorized into seven major lineages: Immune, Endothelial, Epithelial, Stromal, Erythroid, Neural, and Muscle (Figures 1D, S1, and S2/Tables S5 and S6). To substantiate the annotations of novel subtypes, such as macrophages, we performed differential expression analysis followed by functional enrichment analysis. The results confirmed that these clusters possess distinct transcriptomic signatures and biological functions consistent with their assigned identities (Figure S3A).

Cell lineages were subsequently ordered chronologically according to developmental timeline, revealing dynamic shifts in lineage composition across stages (Figure 1E). Projection of cells across stages into a unified developmental trajectory highlighted extensive transcriptomic transitions from embryonic to adult stages. In cardiac and LDM tissues, stromal and muscle cells remained predominant, whereas the liver displayed marked temporal reorganization: erythroid cells dominated during fetal stages but sharply declined after birth, with immune and epithelial lineages progressively expanding (Figure S3B).

During development, complex tissues undergo compositional changes mediated by variations in stem cell proliferation, differentiated cell proportions, and subtype specification [23, 24, 34]. To quantify shifts in cell type and lineage composition during pre‐ and postnatal developmental process, cellular composition changes were assessed by comparing the ratio of cell number between consecutive developmental stages. Muscle, epithelial, and immune lineages progressively expanded during development, while erythroid and stromal lineages decreased (Figures 1F and S3C). These results reflect the coordinated remodeling of tissue architecture through stem/progenitor proliferation, lineage specification, and functional maturation during the transition from embryonic to adult stages.

### Decomposition of transcriptional variance identifies lineage and tissue as primary drivers and pinpoints key developmental transitions

**Figure 2:**
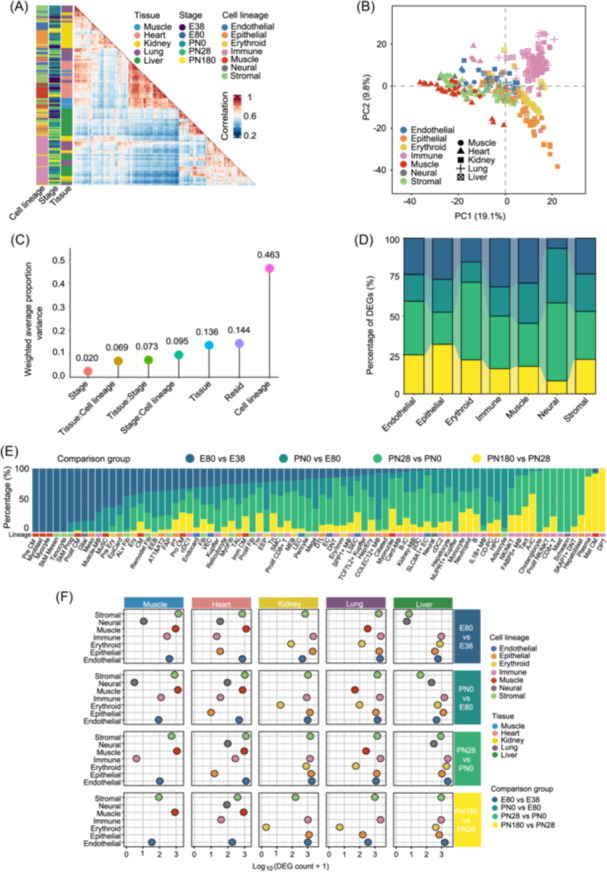
Dynamics of differential gene expression and transcriptional variability across developmental stages in pig. (A) Spearman correlation analysis of the gene expression similarity across cell types, highlighting the relationships between gene expression variability across different cell types, tissues, and stages. (B) Principal component analysis (PCA) of gene expression data, illustrating the major sources of variation in gene expression across the studied tissues and developmental stages. (C) Principal variance component analysis (PVCA) showing the contribution of stage, tissue, cell lineage to gene expression variation. Proportions of differentially expressed genes across stages for each lineage (D) and cell type (E), with colors representing the pair of adjacent time points being compared, revealing the dynamic changes in gene expression during development. (F) Dot plot showing the number of differentially expressed genes (DEGs), represented as log_10_ (DEG count + 1) on the *x*‐axis, across cell lineages (*y*‐axis) in five tissues, comparing adjacent developmental time points, highlighting lineage‐ and tissue‐specific changes in gene expression.

To quantify the contributions of key biological variables to transcriptional heterogeneity, global transcriptomic variance was decomposed. During fetal development, strong correlations were observed between muscle and stromal lineages, as well as among immune cells, indicating shared functional properties during fetal development (Figure 2A). Principal component analysis (PCA) and principal variant analysis (PVCA) further showed that cell lineage and tissue origin are the predominant contributors for the transcriptional heterogeneity, explaining 46.3% and 13.6% of total variance, respectively (Figure 2B,C).

To examine the changes in differentially expressed genes (DEGs) across development (Tables S7 and S8), we conducted differential abundance analysis between consecutive stages within each lineage (Figure 2D), cell type (Figure 2E) and tissue (Figure 2F). Notably, our analysis identified birth (PN0) as a systemic transcriptomic inflection point across multiple lineages, coinciding with the critical physiological adaptations required for extrauterine life. In the neural lineage, DEGs were predominantly found in the comparisons between PN0 (postnatal day 0) versus E80 (embryonic day 80) and PN28 (postnatal day 28) versus PN0 (postnatal day 0), together accounting for over 80% of the total DEGs. The concentration of transcriptional changes at the PN0 transition likely reflects the rapid neural remodeling necessary to process novel sensory stimuli and regulate autonomic functions immediately following birth. Similarly, in the erythroid lineage, more than 50% of the DEGs were observed in the PN28 versus PN0 comparison (Figure 2D). This postnatal surge highlights a crucial regulatory window for erythroid development [35], consistent with the physiological shift from fetal to adult hemoglobin and the adaptation to pulmonary respiration.

Immune cell types (e.g., NK, CD8^+^ T cells) also exhibited DEGs concentrated around birth (Figure 2E). This pattern mirrors the establishment of the neonatal immune system triggered by sudden exposure to environmental antigens and the acquisition of passive immunity. In contrast, myogenic cells (myoblasts, myocytes, SkM Mesen) and tenocytes showed the most significant transcriptional changes earlier in development (E80 vs. E38), indicating that their major phenotypic determination occurs during late embryogenesis rather than at birth.

At the tissue level, endothelial cells were the primary source of DEGs across most tissues in adjacent time‐point comparisons, except for muscle (PN180 vs. PN28). The immune lineage also contributed significantly to DEGs in all tissues except for the heart and muscle (Figure 2F). In the heart and muscle tissues, the main source of DEGs was from stromal and muscle cells, suggesting distinct developmental processes occurring in these tissues during postnatal development. CytoTRACE‐based inference of the tissue/lineage stemness further revealed a progressive decline in developmental potential, particularly within muscle tissue (Figures S4 and S5).

### Regulatory networks drive lineage specification and functional maturation in porcine development

**Figure 3:**
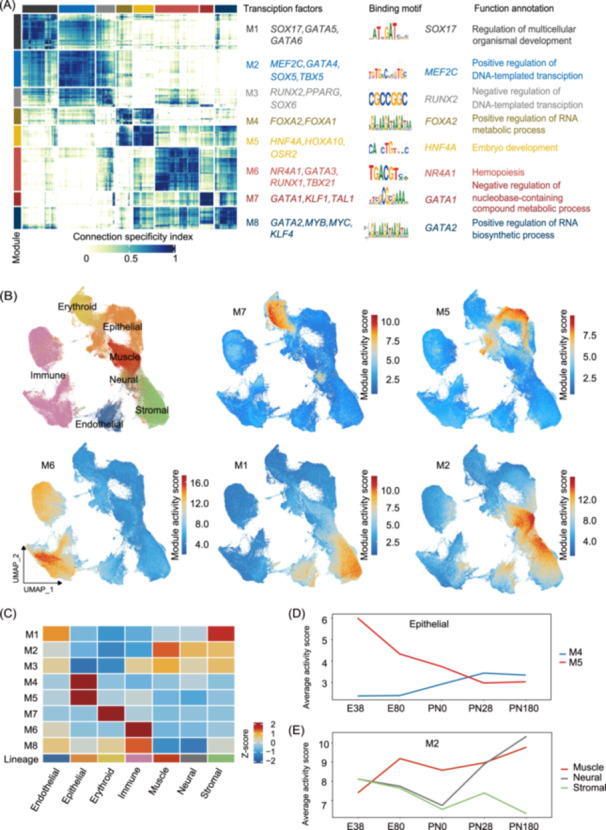
Transcriptional regulon modules orchestrate pig development through temporally ordered and lineage‐specific programs. (A) Heatmap showing the similarity of 568 transcription factor (TF) regulons aggregated into eight major modules, with representative TFs, corresponding binding motifs, and associated functions. (B) UMAP visualization of regulon activity scores from selected modules, demonstrating the spatiotemporal segregation of regulon activities, with distinct enrichment of M1 in stromal cells, M2 in muscle, M5 in epithelial, and M7 in erythroid populations. (C) Heatmap of regulon activity scores (RAS) of the eight transcriptional modules across seven cell lineages. (D) Line plot showing developmental changes in the average RAS of M4 and M5 within the epithelial lineage. (E) Line plot showing developmental changes in the average M2 RAS across the muscle, neural, and stromal lineages.

To systematically characterize the regulatory logic underlying porcine development, we combined high‐dimensional weighted gene co‐expression network analysis (hdWGCNA) with TF regulon inference using pySCENIC. While hdWGCNA identified 15 distinct modules with marked spatiotemporal specificity, spanning early stromal establishment, postnatal metabolic specialization, and immune activation (Figure S6 and Table S9), pySCENIC resolved the upstream TF networks associated with these developmental programs. By identifying 568 regulons organized into eight major modules, we found that distinct lineage identities were associated with characteristic sets of master regulators (Figure 3A). For example, the *SOX17*/*GATA6*‐centered vascular program (M1) and the *MEF2C*/*GATA4*‐centered contractile maturation program (M2) were associated with structural tissue specialization. Likewise, dedicated regulons were linked to adaptive immune programs (*NR4A1*, *GATA3*, and *TBX21* in M6) and erythroid lineage specification (*GATA1* and *KLF1* in M7) (Figures 3A–C and S7). To further evaluate the inferred regulons and confirm that the predicted regulatory links are physically functional, we integrated high‐resolution single‐nucleus ATAC‐seq (snATAC‐seq) data from a recently published study on porcine immune cells [22]. We focused on the M8 module for further examination because it showed immune‐lineage‐specific activity and was therefore most directly comparable to the available snATAC‐seq dataset. This analysis showed significant enrichment of chromatin accessibility at predicted target sites of *KLF4* (2.5‐fold enrichment; *p* < 10^−15^) and *GATA2* (1.6‐fold enrichment; *p* < 0.001) relative to background (Figure S8).

Beyond lineage specification, our analysis showed that developmental progression was accompanied by dynamic temporal shifts in regulon activity. A representative example was observed in the epithelial lineage, where the prenatal M5 module (*HNF4A*/*HOXA10*), associated with embryonic morphogenesis, gave way postnatally to the M4 module (*FOXA1*/*FOXA2*), which was linked to metabolic homeostasis (Figure 3D). In addition, the temporal behavior of shared regulatory programs suggested that common regulon modules were deployed differently across tissues according to developmental context. For example, the contractile M2 module showed a progressive increase in activity within the muscle lineage, whereas in stromal compartments it displayed a biphasic pattern centered around birth, indicating distinct developmental trajectories between these tissues (Figure 3E).

### Spatiotemporal landscape of immune cell development reveals a bifurcated trajectory and tissue‐adapted regulation

**Figure 4:**
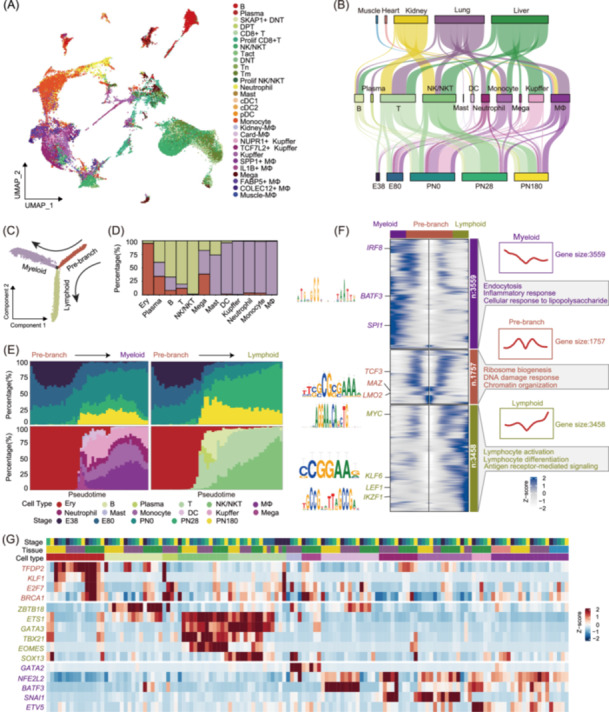
Developmental trajectories reveal the temporal and regulatory coordination of immune lineage differentiation during pig development. (A) UMAP visualization of 29 immune cell subtypes across five tissues and developmental stages; detailed cell type annotations are shown in the right‐hand panel. (B) Sankey diagram illustrating the dynamic changes in the abundance of 11 major immune cell types across tissues and developmental stages. (C) Pseudotime trajectory reconstruction of immune cells using Monocle 2, colored by cell fate branches. (D) Stacked bar chart illustrating the relative distribution of erythroid cells and 11 major immune cell types across the three cell fate branches reconstructed from pseudotime analysis. Each bar corresponds to a cell type, with colored segments indicating the proportion of cells mapped to each cell fate. Branch coloring is consistent with (C). (E) Stacked plots showing the temporal distribution of cells along the pseudotime axis, divided into 100 bins. The plots are split into left and right panels representing the two major trajectories: erythroid‐to‐myeloid (left) and erythroid‐to‐lymphoid (right) differentiation. In each panel, the upper section illustrates the proportion of cells originating from the five developmental stages across pseudotime, while the lower section depicts the composition of erythroid cells and 11 major immune cell types within each stage‐specific pseudotime interval. (F) Transcriptional regulation and functional dynamics of the bifurcated immune trajectory. Left panel: Key TFs and their corresponding binding motifs identified as drivers for each lineage branch (e.g., *IRF8*, *BATF3*, *SPI1* for Myeloid; *TCF3*, *MAZ*, *LMO2* for Pre‐ branch; *KLF6*, *LEF1*, *IKZF1* for Lymphoid). Middle panel: Heatmap visualizing the expression dynamics of branch‐specific gene modules along pseudotime. Rows represent differentially expressed genes clustered by temporal pattern, and columns represent single cells ordered by pseudotime. Right panel: Line graphs illustrate the LOESS‐smoothed expression dynamics of the genes along the pseudotime trajectory (Myeloid ‐> Pre‐ branch ‐> Lymphoid). The number of genes in each cluster is indicated (Gene Size). Boxes list representative Gene Ontology (GO) biological processes enriched in each module, demonstrating that these specific functions follow the visualized activation kinetics during lineage commitment. (G) Dynamic expression of representative immune‐related genes highlighting cell type‐, tissue‐, and lineage‐specific expression patterns.

To systematically decipher the spatiotemporal dynamics and inter‐lineage crosstalk during immune system development, a total of 74,572 immune cells were isolated and analyzed. Unsupervised clustering identified 29 immune cell subtypes, including B cells, plasma cells, multiple T cell subsets (DNT, CD4^+^CD8^+^ T, activated T cells), NK/NKT cells, mast cells, dendritic cells (cDC1, cDC2 and pDC), neutrophils, monocytes, megakaryocytes, Kupffer cells, and several macrophage subtypes (Figure 4A). Immune cells were detected as early as E38, with their abundance and diversity increasing throughout fetal and adult stages (Figure 4B). A clear developmental shift in immune composition was noted, myeloid cells dominated during embryonic stages (E38 and E80), while lymphoid cells significantly increased in proportion after birth (PN0, PN28, and PN180) (Figure 4B). Notable tissue‐specific distribution patterns were observed, macrophages were identified as the only immune cell type detected in muscle and heart tissues, while kidney, lung, and liver exhibited considerably higher immune diversity (Figure 4B).

Pseudotime analysis identified a bifurcated transcriptional trajectory in which erythroid‐like cells occupied the pre‐branch region, followed by two transcriptionally distinct branches enriched for myeloid and lymphoid cells, respectively (Figure 4C and Table S10). Specifically, myeloid cells, including macrophages, monocytes, and dendritic cells, predominantly mapped to one branch, while lymphoid cells (B cells, T cells, and NK cells) were distributed along the other (Figures 4D,E, and S9). This branching topology was consistently observed across tissues and developmental stages, suggesting a conserved transcriptional architecture underlying immune cell diversification (Figure S9).

Key TFs with branch‐specific enrichment and functional specialization were identified (Figure 4F and Table S11) Myeloid commitment was driven by TFs such as *BATF3* [36], *IRF8* [37], and *SPI1* [38], which are associated with endocytosis and phagocytosis. The lymphoid branch was characterized by *LEF1* [39, 40], *KLF6*, *MYC* [41, 42] and *IKZF1* [43], which are involved in differentiation and activation of lymphocytes. Widespread spatiotemporal and tissue‐specific expression patterns were observed among these TFs (Figure 4G). Notably, erythroid regulation showed clear tissue adaptation, with distinct TF usage across developmental stages and organs. In contrast, lymphoid cells combined broadly expressed regulators (e.g., *ETS1* and *GATA3*) with subset‐ or tissue‐restricted factors such as *TBX21*, *EOMES*, and *SOX13* (Figure 4G).

### Cellular dissection of domestic selection signatures implicates neural and muscular lineages as primary targets

**Figure 5:**
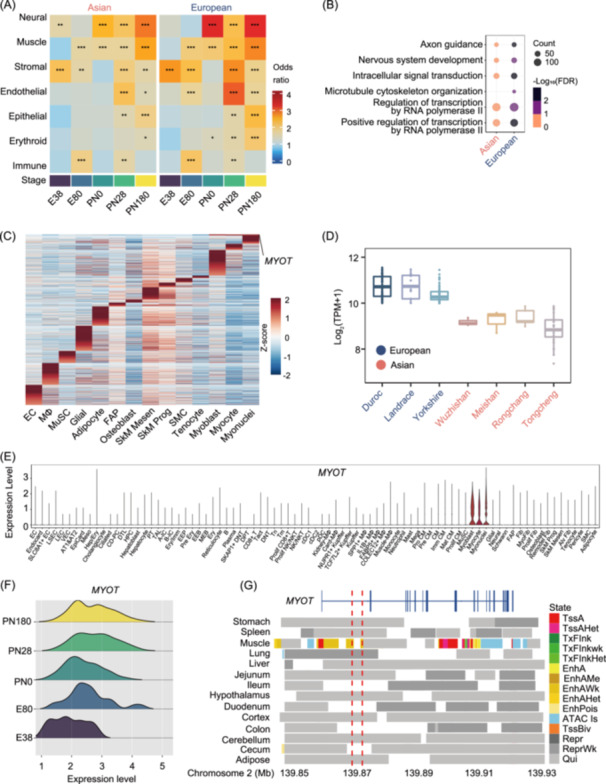
Single‐cell mapping of domestication‐associated regulatory programs highlights *MYOT* as a muscle‐specific target in European pigs. (A) The heatmaps show Fisher's exact test odds ratios (ORs) quantifying the overlap between lineage‐specific gene sets and selective gene sets identified in Eurasian domestic pigs. Heatmap colors represent enrichment ORs. Statistical significance was assessed using Fisher's exact test followed by Benjamini–Hochberg false discovery rate (FDR) correction across all tested lineage–selection comparisons. Asterisks indicate FDR‐adjusted significance levels (*q < 0.05, **q < 0.01, ***q < 0.001). (B) Gene Ontology (GO) terms enriched among selective genes assigned to muscle lineages in Eurasian pigs, with top terms shown to illustrate distinct functional themes between lineages and breeds. (C) Expression heatmap of selective genes in muscle cell types. The abbreviations of cell types are as follows: Endothelial cells (EC), Macrophages (MΦ), Muscle stem cells (MuSC), Adipocytes (Adipocyte), Fibro/adipogenic progenitors (FAP), Osteoblasts (Osteoblast), Skeletal muscle mesenchymal cells (SkM Mesen), Skeletal muscle progenitor cells (SkM Prog), Smooth muscle cells (SMC). (D) *MYOT* expression levels (Log_2_[TPM + 1]) in European (Duroc, Landrace, Yorkshire; *n* = 132 animals) and Asian (Wuzhishan, Meishan, Rongchang, Tongcheng; *n* = 40 animals) pig breeds. Each point represents one individual animal. Statistical significance was assessed using an unpaired Wilcoxon rank‐sum test (two‐sided). *p* < 2.2 × 10^−16^. (E) Violin plot showing the expression level of *MYOT* across cell types. (F) Ridge plot showing *MYOT* expression level in Myonuclei across development stages. (G) Chromatin state landscape at *MYOT* locus across 14 tissues (TssA: Strongly active promoters/transcripts; TssAHet: Flanking active TSS without ATAC; TxFlnk: Transcribed at gene; TxFlnkWk: Weak transcribed at gene; TxFlnKHet: Transcribed region without ATAC; EnhA: Strong active enhancer; EnhAMe:Medium enhancer with ATAC; EnhAWk:Weak active enhancer; EnhAHet:Active enhancer no ATAC; EnhPois:Poised enhancer; ATAC_Is:ATAC island; TssBiv:Bivalent/poised TSS; Repr:Repressed polycomb; ReprWk:Weak repressed polycomb; Qui:Quiescent).

Domestic pigs have undergone strong artificial selection since their divergence from wild boars, [44], represented by dramatic changes in body size, head/body composition and reproductive traits to fulfill the needs of humans [45, 46]. Although selective sweep regions have been extensively mapped at the genomic level [47], the key cell types potentially underlying the phenotypic effects of domestication remain largely unknown. By integrating signatures of positive selection from both Asian and European pig breeds with our spatiotemporal cellular atlas, we identified cell type‐specific selection patterns associated with domestication (Figures 5A, S10, and S11).

Notably, genes under strong selection in both Asian and European domestic pigs were highly enriched in neural, muscular, and stromal lineages (Figure 5A). Muscle‐related selection signatures became prominent from embryonic day 80 (E80) onward (Figure 5A), coinciding with the onset of active myogenic differentiation. Functional enrichment analysis revealed distinct biological processes under selection between Asian and European pigs, including axon guidance, nervous system development, intracellular signal transduction, microtubule cytoskeleton organization and regulation of transcription by RNA polymerase (Figure 5B). These processes reflect adaptations in metabolic regulation and structural integrity, and suggest a potential role of neuroendocrine and vascular functions in mediating the complex trait divergence between European and Asian pigs. Furthermore, quantitative trait locus (QTL) enrichment analysis highlighted the phenotypic divergence between the two groups. In the muscle lineage, genes selected in Asian pigs were strongly linked to fatty acid composition (e.g., arachidic acid to stearic acid ratio and eicosenoic acid to eicosanoic acid ratio). Conversely, genes selected in European pigs were predominantly associated with fat deposition (e.g., leaf fat percentage), lipid metabolism (e.g., myristoleic acid to myristic acid ratio), and body size (e.g., number of ribs) (Figures S12A and S12B).

The most striking phenotypic divergence between European and Asian breeds is meat production [48], which includes growth and carcass traits, meat quality, and the muscle structural integrity underlying these phenotypes. We further investigated the expression of selected genes within muscle‐related cell types. A key selected gene, *MYOT* (Myotilin), which encodes a Z‐disc associated protein critical for actin filament stability and sarcomeric integrity [49], showed the highest expression in myogenic cell types, including myoblasts, myocytes, and myonuclei (Figure 5C). Notably, *MYOT* expression was significantly elevated in European breeds compared to their Asian counterparts at the individual animal level (Figure 5D), as indicated by PigGTEx data [50]. Across all 83 annotated cell types, *MYOT* expression was predominantly restricted to these myogenic lineages and increased progressively during postnatal development (Figure 5E,F). Chromatin state profiling across tissues confirmed an active regulatory landscape at the *MYOT* locus (Figure 5G). Furthermore, phenome‐wide association studies revealed a strong association between *MYOT* variants and meat production traits (Figure S12C and Table S12). Collectively, these genomic and phenotypic data suggest that *MYOT* represents a candidate target of selection that may contribute to differences in meat production‐related phenotypes between European and Asian pig breeds.

### Transcriptomic conservation and divergence between porcine and human prenatal cell lineages

**Figure 6:**
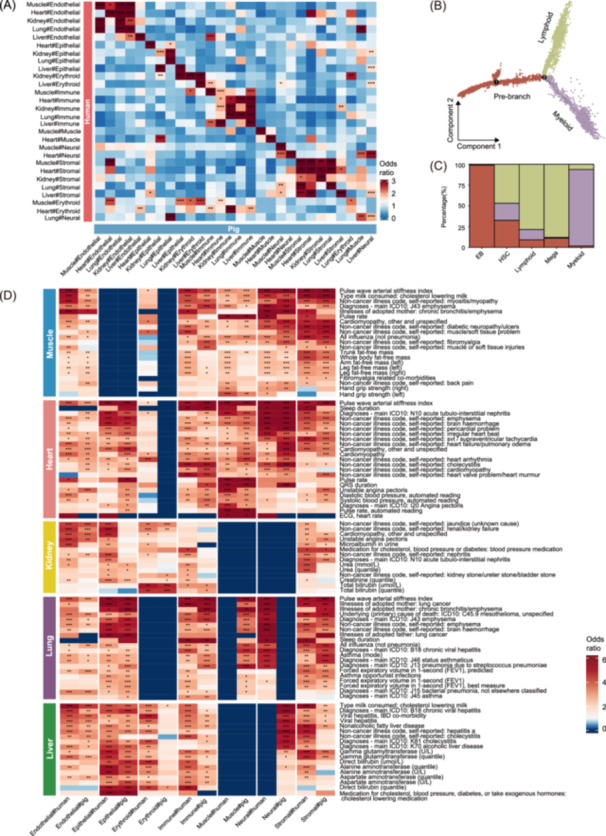
Cross‐species single‐cell mapping reveals conserved lineage specification and disease relevance. (A) Evaluation of transcriptomic conservation across major cell lineages between human and pig. The heatmap displays Fisher's exact test odds ratios (ORs) quantifying the overlap of the top 200 highly expressed orthologous genes between matched species cell lineages. Statistical significance was assessed using Fisher's exact test followed by Benjamini–Hochberg FDR correction across all tested lineage–lineage comparisons. Asterisks indicate FDR‐adjusted significance levels (*q < 0.05, **q < 0.01, ***q < 0.001). (B) Pseudotime trajectory reconstruction of immune cell development using Monocle 2. The trajectory is colored by distinct cell fate branches, illustrating the developmental bifurcation points. (C) Stacked bar chart showing the lineage composition of the cell fate branches identified in (B). Cell‐type abbreviations in (C) are as follows: Megakaryocytes (Mega), Hematopoietic stem cells (HSC), Erythroblasts (EB), Lymphoid cells (Lymphoid), Myeloid cells (Myeloid). (D) Enrichment analysis of human disease‐associated genes derived from GWAS across matched human and porcine cell lineages categorized by tissue. The heatmap shows the enrichment strength, represented by odds ratios, of human disease risk genes expressed in corresponding tissues and cell lineages of both species, highlighting conserved disease‐gene expression patterns in stromal and immune cells. Statistical significance was determined by Fisher's exact test followed by Benjamini–Hochberg FDR correction within each tissue across all tested phenotype–cell lineage pairs. Asterisks indicate FDR‐adjusted significance levels (*q < 0.05, **q < 0.01, ***q < 0.001).

To evaluate the translational relevance of our porcine atlas and establish cross‐species mapping, we integrated our dataset with publicly available human prenatal single‐cell references, as matched postnatal human samples were limited. Following rigorous data harmonization and annotation of conserved lineages (Figures S13 and S14), we systematically quantified the transcriptomic similarity between porcine and human cell lineages across tissues. Our analysis revealed a robust conservation of cell lineage‐specific gene expression between human and pig, particularly within the endothelial, stromal, and immune cell lineages (Figure 6A). We also explored the shared and species‐specific gene expression programs and highlighted conserved TFs within each cell type between pig and human (Table S13). To further explore the conservation of immune transcriptional organization across species, we used Monocle 2 to reconstruct the pseudotime trajectory of human immune‐related cells and identified a bifurcated transcriptional trajectory (Figure 6B,C). Consistent with the pattern observed in prenatal pig cells (Figure 4C,D), this trajectory contained two transcriptionally distinct branches enriched for lymphoid and myeloid populations, respectively. These results suggest a conserved transcriptional architecture associated with immune cell diversification across species.

Furthermore, to assess the utility of the pig single‐cell atlas for modeling the cellular basis of human diseases, we mapped human disease‐associated genes (derived from genome‐wide association studies (GWAS) data) to their porcine homologs and evaluated their enrichment across tissues and cell lineages. We observed a significant conservation of disease gene expression patterns, particularly within stromal and immune cells (Figure 6D), indicating that pig models are suitable to explore the etiology in these cell lineages. Collectively, these findings provide a molecular blueprint for utilizing the prenatal pig as a model for analyzing human development and disease, while also delineating the specific boundaries of its applicability.

## DISCUSSION

The pig is recognized as a significant agricultural animal and an invaluable biomedical model. Investigation of its genetic regulation and physiological mechanisms is therefore crucial for both human health and agricultural trait improvement. In this study, we constructed a dynamic single‐cell transcriptomic atlas of porcine development, mapping 83 cell types across five major tissues and developmental stages. This substantially expands the existing cellular landscape and enables a deeper understanding of developmental and regulatory dynamics in pigs. By integrating single‐cell and single‐nucleus RNA sequencing data across multiple tissues, we systematically mapped cell‐type diversity and delineated lineage hierarchies underlying organogenesis. This dataset bridges a critical gap between agricultural and biomedical research, offering a valuable reference for investigating gene regulation, tissue specialization, and evolutionary adaptation in a species of both economic and translational significance.

We identified pronounced transcriptional reprogramming around birth (PN0), corresponding to a major physiological transition from fetal to postnatal life [51, 52]. This stage was accompanied by large‐scale shifts in cellular composition—such as the replacement of erythroid dominance by immune and epithelial expansion—and functional remodeling of shared cell types across tissues [53]. These findings underscore the coordinated interplay between cell proliferation, differentiation, and metabolic adaptation during postnatal tissue maturation.

To gain a systems‐level understanding of the transcriptional programs underlying porcine organ development, we integrated co‐expression (hdWGCNA) [54] and regulatory network analyses (SCENIC regulons) [55]. hdWGCNA delineated modules of genes exhibiting correlated expression patterns across tissues and developmental stages, reflecting coordinated transcriptional programs. SCENIC reconstructed TF‐centered regulons that define direct regulatory dependencies. Integrating these two layers provides mechanistic insight into how lineage commitment and tissue identity are encoded at the transcriptional level. For example, erythroid‐associated modules were enriched for regulons driven by *GATA1* and *KLF1* [56], whereas hepatocyte modules were dominated by *HNF4A*‐ and *CEBPA*‐regulated gene sets [57, 58]. This concordance demonstrates that hdWGCNA modules are not merely statistical constructs but represent biologically coherent transcriptional circuits underpinned by defined regulatory control. Together, these results highlight key TFs that drive lineage specification and functional maturation during pig development.

Cross‐tissue analysis of porcine immune cells revealed a pronounced maturation gradient, tissue‐specific specialization, and a conserved bifurcated transcriptional trajectory [59]. Distinct immune cell compositions were observed across organs, with macrophages predominating in muscle and heart [60, 61, 62], whereas the kidney, lung, and liver displayed greater immune diversity [63, 64, 65]. Integration of regulon activity patterns uncovered coordinated activation between immune and non‐immune compartments. The M6 and M8 regulons—associated with lymphoid differentiation and cellular stress responses—were co‐activated in immune and endothelial cells, highlighting a coordinated maturation process between vascular and immune systems [66, 67]. This crosstalk suggests that immune development in pigs is not solely driven by intrinsic lineage programs but also influenced by stromal and endothelial interactions that shape immune maturation, trafficking, and tissue homing. Key TFs such as *LEF1* [40], *MYC* [41, 42], *BATF3* [36], *IRF8* [37], and *GATA* family members [68, 69] displayed conserved activity across species, supporting evolutionary conservation of immune developmental pathways. However, species‐specific nuances in expression dynamics suggest unique adaptations of porcine immunity, with implications for breeding and biomedical applications, including xenotransplantation.

By integrating cell‐type‐resolved transcriptomic maps with genomic selective sweep data from Asian and European domestic pigs, we provide a cellular framework for interpreting the phenotypic effects of domestication. Our results reveal that selective sweep signals are not evenly distributed across all cell types or tissues. Instead, certain tissues and cell types exhibit a higher enrichment of genes in these selected regions. Genes under selection were particularly enriched in muscle, neural, and stromal cell lineages, suggesting that artificial selection has preferentially targeted these cell populations to modulate traits such as growth rate [70], meat quality, and fat deposition.

Notably, integrative multi‐omic and single‐cell analyses identified a muscle‐specific enhancer of the *MYOT* gene as a target of artificial selection that likely contributes to meat‐quality divergence between Asian and European pig breeds. *MYOT* has been implicated in myogenesis [71, 72], and its selection in domesticated pigs may represent a molecular mechanism supporting differences in muscle properties and production traits among modern breeds. By mapping *MYOT*'s chromatin state of this regulatory element together with *MYOT* expression patterns in muscle cells, we provide a link between genetic selection and functional changes at the cellular level. These findings contribute to the broader understanding of domestication and selective breeding mechanisms and offer valuable information for potentially improving breeding practices.

Cross‐species comparison between pig and human further revealed substantial conservation of cell types and lineage‐specific regulatory features, underscoring the translational relevance of the pig as a biomedical model. By integrating our porcine atlas with publicly available human prenatal single‐cell references, we established cross‐species mapping across conserved endothelial, stromal, and immune lineages, and observed robust conservation of lineage‐specific gene expression patterns between the two species [8, 73]. We further identified shared and species‐specific gene expression programs, as well as conserved TFs within corresponding cell types, highlighting both common developmental principles and lineage‐specific divergence [74, 75]. Notably, reconstruction of pseudotime trajectories in human and prenatal pig immune‐related cells revealed a conserved bifurcated transcriptional trajectory, characterized by two transcriptionally distinct branches enriched for lymphoid and myeloid populations. This cross‐species consistency suggests an evolutionarily conserved transcriptional architecture associated with immune cell diversification [76, 77]. Moreover, the significant conservation of disease‐associated gene expression patterns, particularly in stromal and immune cells, further underscores the value of the reference pig cell atlas as a resource for biomedical research. At the same time, species‐specific differences identified in our analyses define important boundaries for the application of pig models in studies of human development and disease.

While this study encompasses multiple tissues and developmental stages, several limitations should be noted. First, the sampling scope was restricted to the Bama pig breed. As developmental timing and cellular composition can vary significantly among breeds, our developmental trajectory findings may not be fully generalizable to all porcine varieties. Future studies could further validate these findings by extending the sampling window and including additional pig breeds to increase data diversity. Second, our analytical framework involved integration of diverse datasets, including both single‐cell (scRNA‐seq) and single‐nucleus (snRNA‐seq) RNA sequencing platforms, which introduces inherent technical complexities. Although Harmony was utilized to correct for batch effects across sources, methods, platforms, and individuals, balancing comprehensive batch mixing with the preservation of true biological variation remains a persistent challenge. Specifically, integrating scRNA‐seq and snRNA‐seq data is confounded by intrinsic biological biases, such as disparities in cytoplasmic versus nuclear RNA abundance and differential detection rates of non‐polyadenylated transcripts. These technical disparities can directly impact the resolution and accuracy of cell type annotation. This is particularly critical for dynamic populations such as immune cells, where activation states are often characterized by nuanced transcriptional shifts that may be obscured by the absence of cytoplasmic transcripts in snRNA‐seq data. Consequently, future single‐cell atlas efforts will require increasingly rigorous quantitative metrics to continuously evaluate integration success and ensure rare biological signals are not over‐corrected. Finally, although we identified links between selective sweep regions and specific cell types, these findings are still in their preliminary stages. Further functional experiments, such as CRISPR interference or ATAC‐seq [78], are necessary to validate the causal relationships between candidate regulatory elements and specific phenotypic traits.

Together, this study establishes a foundational reference for the single‐cell transcriptomic landscape of pig development. By integrating spatiotemporal transcriptional dynamics, gene regulatory networks, selection signatures, and cross‐species comparisons, we provide mechanistic insights into how cellular diversity and molecular coordination shape organogenesis and trait evolution. The atlas also offers a valuable comparative resource for biomedical research, highlighting conserved regulatory frameworks while exposing pig‐specific developmental features that bridge agricultural and translational biology.

## CONCLUSION

In conclusion, our study establishes a cross‐tissue single‐cell transcriptomic atlas of porcine development. We reveal that coordinated stem/progenitor cell proliferation, lineage specification, and maturation underlie organogenesis, governed by dynamic transcriptional networks. Trajectory analysis further uncovered a conserved transcriptional bifurcation pattern associated with immune cell diversification. Integration with population genomics identified a muscle enhancer near the *MYOT* gene, targeted by selection and linked to meat quality divergence. Cross‐species comparisons confirm substantial cellular conservation with humans. Collectively, this atlas serves as a key resource for understanding mammalian development, informing precision breeding strategies, and advancing the pig as a biomedical model.

## MATERIALS AND METHODS

### Collection of animal tissues

The experimental animals were selected from a total of 15 healthy Bama pigs at key developmental stages: embryonic day 38 (E38), embryonic day 80 (E80), postnatal day 0 (PN0), postnatal day 28 (PN28), and postnatal day 180 (PN180). Following a 12‐h fast, the subjects were bled and slaughtered in accordance with traditional humane methods. Five tissues were obtained from the liver, lung, kidney, heart and LDM, respectively, within 30 min of the subject's demise. The tissue samples were immediately placed into sterile cold PBS at 4°C and subsequently transported to the tissue culture laboratory for single‐cell/nucleus suspension preparation. Each tissue was dissociated and digested independently.

### Single‐Cell/Nucleus library preparation and sequencing

To comprehensively capture cellular and nuclear transcriptomes, lung and kidney samples were processed for scRNA‐seq, heart and skeletal muscle for snRNA‐seq, and liver for both scRNA‐seq and snRNA‐seq. For each tissue and developmental stage, mixed samples from three biological replicates were processed through two technical repeats independently. Single cells/nuclei were captured using the DNBelab C4 microfluidic platform (MGI Tech Co., Ltd.), with subsequent in‐situ lysis, barcoding, and reverse transcription occurring within nanowells. Sequencing libraries were constructed from captured material using the DNBelab C Series Single‐Cell Library Prep Kit (MGI) per manufacturer protocols, encompassing cDNA synthesis/pre‐amplification (for scRNA‐seq/snRNA‐seq), fragmentation, end repair, a‐tailing, adapter ligation, and sample indexing PCR for unique dual indices (UDI) incorporation. Pooled libraries were quantified, qualified, and subjected to high‐throughput paired‐end 100 bp (PE100) sequencing on the DNBSEQ‐T7 platform (MGI), involving DNA Nanoball (DNB) generation on patterned nanoarrays and combinatorial probe‐anchor synthesis chemistry.

### Raw data processing, quality control and doublet removal

The raw sequencing data from scRNA‐seq/snRNA‐seq were processed using the DNBelab C Series scRNA analysis pipeline to generate UMI‐by‐gene expression matrices [79]. Reads were aligned to the pig reference genome Sscrofa11.1 (Ensembl release 110) [80]. To ensure consistent quantification across both single‐cell and single‐nucleus libraries, genomic mapping was performed with parameters set to include intronic reads, thereby capturing both mature mRNA and nascent pre‐mRNA. Cells/nuclei were retained if they met the following quality‐control thresholds: detected gene count between 200 and 8000, and percentage of mitochondrial transcripts < 30% (mitochondrial genes considered: *ATP6*, *ATP8*, *COX1*, *COX2*, *COX3*, *CYTB*, *ND2*, *ND3*, *ND4*, *ND4L*, *ND5*, *ND6*). The pig *ND1* gene was excluded from mitochondrial filtering due to high sequence variability. Within‐sample doublets were identified and removed using Seurat's DoubletFinder function with default parameters [81].

### Cell clusters identification and global variance decomposition

Seurat (v4.4) was used to perform unsupervised clustering [82]. Libraries from the same tissue were merged and underwent normalizing and scaling. Harmony (v1.2.3) [83] was used to correct four batch effects (sources, methods, platforms, and individuals) with the resetting parameters (Lambda = 1, Theta = 0.5) (Figure S15). Variable genes were determined using Seurat's FindVariableFeatures with default parameters (selection.method = “vst”, nfeatures = 2000). Clusters were identified via the FindClusters function (0.6 < resolution < 1.2) implemented in Seurat using the top 30 principal components and subsequently visualized using the RunUMAP function (reduction = “harmony”). Clusters were annotated manually using canonical marker genes from the literature. Global transcriptional variance was decomposed using PCA together with principal variance component analysis (PVCA) or mixed‐effects modeling to estimate the contributions of factors such as lineage, tissue of origin, developmental stage.

### Cell composition analysis

To quantify changes in cell‐type/cell lineage composition across development, the relative proportions of cell types/cell lineages were computed for developmental stage. Changes in composition between adjacent stages (e.g., PN0 vs. E80) were compared. Point size in the plot reflects the geometric mean of the two stage‐specific average proportions.

### Identification of DEGs across groups

DEGs were identified using a Presto‐based implementation of the FindAllMarkers functions in Seurat, which applies a Wilcoxon rank‐sum test for differential expression analysis. For each comparison, genes were required to meet the following thresholds: expression in at least 10% of cells in either group (min.pct = 0.10) and an absolute Log_2_ fold change ≥ 0.25 (logfc.threshold = 0.25). Differential expression analyses were performed separately for each cell type, comparing cells from different developmental stages as defined by the orig.ident metadata field. All comparisons were conducted as unpaired analyses, treating individual cells as independent observations, without explicit pairing of the same individuals across developmental stages. *P*‐values were adjusted for multiple testing using the Bonferroni correction, and genes with an adjusted *p* < 0.05 were considered statistically significant.

### Gene regulatory network analysis

Gene regulatory network inference and regulon identification were performed using the Python package pySCENIC (v.0.12.1) with the default parameters [84]. Filtered raw read‐count matrices were used as input after retaining cells with 200–8,000 detected genes. A curated list of pig TFs was generated by orthology mapping with OrthoFinder, using human TF annotations as the reference, and supplied to GRNBoost2 to infer TF–target co‐expression modules [85]. Candidate regulons (TF‐centered regulatory units) were subsequently refined by motif enrichment analysis around transcription start sites (TSS) using cisTarget databases (https://resources.aertslab.org/cistarget/), retaining direct TF targets while excluding indirect associations. For motif analysis in pig, a custom RcisTarget database was constructed with ‘create_cistarget_motif_databases. py‘ based on the Ensembl release 109 annotation of the Sscrofa11.1 reference genome. Feather ranking databases were built for two genomic region definitions: (i) −2500 bp to +500 bp relative to each TSS and (ii) ±10 kb around each TSS. TF binding motifs were derived by mapping human motifs from the SCENIC v10 public motif collection (https://resources.aertslab.org/cistarget/motif_collections/v10nr_clust_public/snapshots/) to pig orthologues, using high‐confidence one‐to‐one orthology relationships obtained via Ensembl BioMart. Specifically, the analysis consists of three main steps: first, arboreto_with_multiprocessing.py is used with the GRNBoost2 method to infer co‐expression modules between TFs and their target genes, with key parameters including ‐‐method grnboost2 to specify the algorithm; second, pyscenic ctx performs cisTarget motif enrichment analysis to distinguish direct from indirect regulatory interactions by testing whether target gene promoters contain the corresponding TF binding motifs, with key parameters including ‐‐annotations_fname to provide motif annotation files, ‐‐expression_mtx_fname to specify the expression matrix, and ‐‐no_pruning to skip intermediate pruning steps; third, pyscenic aucell calculates regulon activity scores per cell, evaluating the relative activity of each regulon based on the enrichment of its target genes among the top‐expressed genes in each cell [86], yielding an RAS matrix comprising 568 regulons. The Connection Specificity Index (CSI) serves as a metric to evaluate the degree of connectedness between regulons. A high CSI between two regulons suggests that they tend to co‐regulate downstream genes and function together in specific biological processes. Using the AUCell‐derived regulon activity matrix, CSI scores were calculated for all regulon pairs. Subsequently, hierarchical clustering with default parameters was performed on the CSI matrix to delineate modules of co‐regulated regulons. To identify modules of co‐regulated TFs/regulons, regulon–regulon relationships were summarized using the Connection Specificity Index (CSI), and the resulting CSI matrix was hierarchically clustered (Euclidean distance) to delineate regulon modules; these were further consolidated into eight major modules for downstream interpretation.

### Function enrichment analysis

Gene Ontology (GO) analysis was performed using the clusterProfiler (v4.14) package [87]. The GO terms of selected genes were enriched in the database “org.Ss.eg.db” using “enrichGO” function, with a threshold parameter of “pvalueCutoff = 0.05”. Benjamini–Hochberg (BH) method was used for the multiple test adjustment.

### High‐dimensional weighted correlation network analysis

We performed high‐dimensional weighted gene co‐expression network analysis (hdWGCNA) as follows [88]. Initially, genes expressed in at least 5% of cells were selected as input, a filtering step that excluded rarely detected genes which would otherwise introduce noise into network construction. The MetacellsByGroups function was then used to create a metacell gene expression matrix with parameters *k* = 25 and min cells = 10, where metacells were constructed using K‐nearest neighbors (KNN) based on biological similarity. This metacell averaging approach aggregates expression profiles over transcriptionally similar cells, making co‐expression relationships more robust and comparable to traditional WGCNA applied to bulk RNA‐seq data, with the k parameter specifying the number of neighbors for aggregation. For pseudobulk analysis, a *K* value of 20 with a maximum of 10 shared neighbors was selected for an average of 5000 cells per population, balancing local structure capture with noise minimization in sparse data; pseudobulk aggregation effectively mimics bulk RNA‐seq profiles to facilitate cross‐condition comparisons.

Subsequently, the TestSoftPowers function was applied to determine the optimal soft power, and the co‐expression network was built using the ConstructNetwork function. Soft‐thresholding with power β strengthens strong correlations while suppressing weak ones to achieve a scale‐free topology. The optimal soft‐thresholding power (*β*) was selected by evaluating the scale‐free topology fit index, aiming for the lowest power at which the network closely approximated a scale‐free structure. *β* values between 6 and 10 were determined as optimal for both pseudobulk and cell cluster‐based analyses, as the scale‐free fit index *R* ^2^ measures adherence to a scale‐free distribution, a hallmark of biological networks. Module eigengenes and connectivity were computed with default parameters. Module eigengenes represent the first principal component of each module, capturing the overall expression pattern across cells.

Module connectivity (kME), which measures the correlation between a gene's expression profile and its module eigengene, was used to identify hub genes, with high kME values indicating a central role within the module; kME values near 1 specifically indicate core module members. The top 25 hub genes were subsequently determined using the GetHubGenes function, as hub genes are considered key regulators of their respective modules.

We then ran the UMAP algorithm on the hdWGCNA topological overlap matrix (TOM) using the RunModuleUMAP function and visualized the UMAP co‐expression network with the ModuleUMAPPlot function. The TOM captures both direct and indirect co‐expression similarities between genes, and UMAP projection reveals module‐level organization as distinct clusters of genes with high topological overlap. Transcriptomic modules were detected via hierarchical clustering of the TOM, which uses 1‐TOM as a distance metric to iteratively merge similar genes into modules. Module eigengenes, defined as the first principal component of each module, were calculated; these eigengenes can be correlated with cell metadata (e.g., cell type, treatment condition, or pseudotime) to link co‐expression modules to biological phenotypes. Gene ontology enrichment analysis of genes in each module was performed to see what biological processes might be associated with each module. This analysis was done, and results visualized using ClusterProfiler.

### Pseudotime trajectory analysis

The Monocle2 (v2.24.0) was used to infer the state transition of the cell types/subtypes [89]. The UMI count matrix of the cells was used to create the CellDataSet object and then filter out the genes expressed in fewer than 10 cells to enhance the stability of the trajectory. The genes with *q* < 0.01 were identified as DEGs using the differentialGeneTest function and sorted by *q* using the setOrderingFilter function. The pseudotime trajectory was constructed by the DDRTree algorithm using the default parameters. The initial point of the trajectory was manually selected according to developmental potential and biological background, e.g. Start from naive immune cells or muscle progenitor cells. The dynamic expression changes of selected marker genes in pseudotime were visualized by the plot_pseudotime_heatmap functions.

### CytoTRACE score

The CytoTRACE algorithm is developed by Gulati et al. to capture, smooth, and calculate the expression level of genes that are most highly correlated with single‐cell gene counts with scRNA‐Seq data. We performed CytoTRACE [90] by using R package CytoTRACE (v0.3.3). CytoTRACE scores range from 0 to 1, while higher scores indicate higher stemness (less differentiation) and vice versa.

### Integration with selective sweep signals and chromatin state data

Selective sweep signals identified in European and Asian domestic pigs and chromatin‐state annotations were obtained from published datasets [91]. In that study, signatures of selection were identified using *F*_*ST*_ analysis by comparing genome‐wide variation among European domestic pigs, Asian domestic pigs, and their respective wild boar populations (sample sizes, breeds, and specific score thresholds are detailed in the original publication). The genomic regions identified as selective sweeps in the European and Asian domestic pig lineages from this resource were used for our subsequent integration analysis. Chromatin states considered included promoters (TssA, TssAHet, TssBiv), TSS‐proximal transcribed regions (TxFlnk, TxFlnkWk, TxFlnkHet), enhancers (EnhA, EnhAMe, EnhAWk, EnhAHet, EnhPois), repressed regions (Repr, ReprWk) and quiescent regions (Qui) across 14 tissues (stomach, spleen, muscle, lung, liver, jejunum, ileum, hypothalamus, duodenum, cortex, colon, cerebellum, cecum and adipose). For integration with selective sweeps, a gene was considered to overlap a sweep region if any part of its defined genomic interval intersected the region. To test whether sweep‐associated genes were preferentially represented in lineage‐specific transcriptional programs, we first defined lineage‐stage‐specific gene sets from tissue‐level differential expression results by retaining genes with LogFC > 0 and *p*.adjust < 0.05, and then selecting the top 500 genes ranked by LogFC for each lineage‐stage group. For each lineage‐stage group, enrichment against the European and Asian sweep‐associated gene sets was assessed independently using Fisher's exact test. Contingency tables were constructed from the numbers of overlapping genes, lineage‐specific‐only genes, sweep‐only genes, and genes in neither category, using a background universe of 27,466 genes. Genomic intersections between genes and the 15 genomic features were performed using bedtools.

### Association analysis of human GWAS traits with pig cell lineages

To evaluate the relevance of the pig single‐cell atlas for modeling human diseases, we analyzed the enrichment of human disease‐associated genes within porcine cell lineages. Human disease‐associated genes derived from GWAS were obtained from the UK Biobank database [92] and published studies. To enable cross‐species comparison, porcine (*Sus scrofa*) gene symbols were mapped to their human orthologs using the babelgene R package (v22.9), excluding genes without one‐to‐one orthologs. We then quantified the overlap between these disease‐associated gene sets and the converted human‐orthologous cell lineage‐specific marker lists stratified by tissue. Fisher's exact test was performed to calculate the odds ratio (OR) and nominal *p*‐value for each phenotype–cell lineage pair. To correct for multiple comparisons, *p* was adjusted using the Benjamini–Hochberg false discovery rate (FDR) method within each tissue. FDR‐adjusted *p*‐values were used for significance annotation on the heatmap, with asterisks indicating *q < 0.05, **q < 0.01, and ***q < 0.001. The heatmap color scale represents OR values.

## AUTHOR CONTRIBUTIONS

**Rong Zhou**: Funding acquisition; writing—original draft; project administration; writing—review and editing. **Zishuai Wang**: Writing—original draft; writing—review and editing; conceptualization; visualization; formal analysis. **Chenghao Hu**: Visualization; formal analysis. **Shuhan Deng**: Data curation; formal analysis; visualization. **Changyun Cai**: Formal analysis. **Yanfang Wang**: Funding acquisition. **Shang‐Tong Li**: Funding acquisition; investigation; writing—original draft; writing—review and editing. **Lijing Bai**: Investigation. **Kui Li**: Funding acquisition; investigation; project administration. All authors have read the final manuscript and approved it for publication.

## CONFLICT OF INTEREST STATEMENT

The authors declare no conflicts of interest.

## ETHICS STATEMENT

All experiments involving animals were conducted according to the ethical policies and procedures approved by the ethics committee of the Institute of Animal Sciences, Chinese Academy of Agricultural Sciences (Beijing, China; approval number IAS2022‐58).

## Supporting information


**Figure S1:** UMAP visualization colored by seven annotated cell lineages.
**Figure S2:** Dot plot showing specific markers for cell types in the heart (A), muscle (B), lung (C), kidney (D), and liver (E).
**Figure S3:** GO enrichment analysis of macrophage subtypes and developmental dynamics of cell type composition.
**Figure S4:** Dynamics of differentiation potential across tissues and development.
**Figure S5:** Lineage‐Specific Dynamics of Differentiation Potential across Development.
**Figure S6:** Spatiotemporally coordinated gene co‐expression modules reveal lineage‐ and tissue‐specific regulatory programs during pig development.
**Figure S7:** Enrichment of Gene Ontology (GO) Biological Processes in transcription factor (TF) co‐expression modules.
**Figure S8:** Chromatin accessibility enrichment analysis for predicted *KLF4* and *GATA2* regulon targets within the M8 immune‐lineage module.
**Figure S9:** Composition of the immune cell trajectory across tissues and developmental stages.
**Figure S10:** Analysis of quantitative specificity and selection in gene datasets from Eurasian pigs.
**Figure S11:** Association between cell type‐specific gene sets and selective signatures in Eurasian pigs.
**Figure S12:** QTL enrichment analysis of domestication regions in Asian and European pig breeds and association analysis of the *MYOT* gene.
**Figure S13:** Composition of the human immune cells and erythroid cells trajectory across tissues.
**Figure S14:** Cross‐species UMAP visualization of five tissues.
**Figure S15:** Integration and evaluation of multi‐tissue single‐cell data with the Harmony algorithm.

**Table S1:** Sample metadata.
**Table S2:** Cell numbers per sample.
**Table S3:** A collection of the marker genes used for each cell type annotation.
**Table S4:** Differential expression of the marker genes used for each cell type annotation.
**Table S5:** Cell numbers of main lineage per sample.
**Table S6:** Number of subtypes in each main lineage.
**Table S7:** A collection of differentially expressed genes for each cell type.
**Table S8:** Differential gene expression test results of the adjacent time points.
**Table S9:** GO enrichment results of each module identified by WGCNA analysis.
**Table S10:** A collection of marker genes for clusters identified in pseudotime analysis of immune cells.
**Table S11:** GO enrichment results of stage‐specifically expressed genes during pseudotime analysis of immune cells.
**Table S12:** Phenome‐wide association results for MYOT.
**Table S13:** Human and pig shared genes for conserved cell types.

## Data Availability

The raw sequence data reported in this paper have been deposited in the Genome Sequence Archive in the National Genomics Data Center [93], China National Center for Bioinformation/Beijing Institute of Genomics, Chinese Academy of Sciences (GSA: CRA037795), which are publicly accessible at https://ngdc.cncb.ac.cn/gsa/browse/CRA037795. The code is publicly available on GitHub at: https://github.com/ZhouR-Lab/Pig_atlas. Supplementary materials (figures, tables, graphical abstract, slides, videos, Chinese translated version and update materials) may be found in the online DOI or iMeta Science (http://www.imeta.science).
