# Supplementary material for: A spatiotemporal single‐cell atlas of porcine development reveals regulatory dynamics and cellular targets of domestication
Source: Imeta. 2026 Jun 4;5(3):e70135. doi: 10.1002/imt2.70135 (PMC13377417; doi:10.1002/imt2.70135)
Supplement: Supplementary file 1 — Figure S1: UMAP visualization colored by seven annotated cell lineages. Figure S2: Dot plot showing specific markers for cell types in the heart (A), muscle (B), lung (C), kidney (D), and liver (E). Figure S3: GO enrichment analysis of macrophage subtypes and developmental dynamics of cell type composition. Figure S4: Dynamics of differentiation potential across tissues and development. Figure S5: Lineage‐Specific Dynamics of Differentiation Potential across Development. Figure S6: Spatiotemporally coordinated gene co‐expression modules reveal lineage‐ and tissue‐specific regulatory programs during pig development. Figure S7: Enrichment of Gene Ontology (GO) Biological Processes in transcription factor (TF) co‐expression modules. Figure S8: Chromatin accessibility enrichment analysis for predicted KLF4 and GATA2 regulon targets within the M8 immune‐lineage module. Figure S9: Composition of the immune cell trajectory across tissues and developmental stages. Figure S10: Analysis of quantitative specificity and selection in gene datasets from Eurasian pigs. Figure S11: Association between cell type‐specific gene sets and selective signatures in Eurasian pigs. Figure S12: QTL enrichment analysis of domestication regions in Asian and European pig breeds and association analysis of the MYOT gene. Figure S13: Composition of the human immune cells and erythroid cells trajectory across tissues. Figure S14: Cross‐species UMAP visualization of five tissues. Figure S15: Integration and evaluation of multi‐tissue single‐cell data with the Harmony algorithm. [file IMT2-5-e70135-s001.docx]

**Supporting information to**

**A spatiotemporal single-cell atlas of porcine development reveals regulatory dynamics and cellular targets of domestication**

**Running title: Cross-tissue single-cell transcriptomic atlas of porcine development**

Rong Zhou^1,2#^*, Zishuai Wang^3#^, Chenghao Hu^1#^, Shuhan Deng^4#^, Changyun Cai^1,2^, Yanfang Wang^1^, Shang-Tong Li^4,5^*, Lijing Bai^3^*, Kui Li^3^*

^1^The State Key Laboratory of Animal Biotech Breeding, Institute of Animal Science, Chinese Academy of Agricultural Sciences, Beijing 100193, China

^2^College of Agriculture and Biology, Liaocheng University, Liaocheng 202059, China

^3^Shenzhen Branch, Guangdong Laboratory for Lingnan Modern Agriculture, Genomics Institute at Shenzhen, Chinese Academy of Agricultural Sciences, Shenzhen 518000, China

^4^Glbizzia Biosciences Co., Ltd, Beijing 102609, China

^5^MOA Key Laboratory of Animal Virology, Center for Veterinary Sciences, College of Animal Sciences, Zhejiang University, Hangzhou 310058, China

^#^These authors contributed equally: Rong Zhou, Zishuai Wang, Chenghao Hu, Shuhan Deng

*Correspondence: [likui@caas.cn](mailto:zhourong03@caas.cn) (Kui Li); [zhourong03@caas.cn](mailto:zhourong03@caas.cn) (Rong Zhou); [bailijing@caas.cn](mailto:bailijing@caas.cn) (Lijing Bai); shangtong.li@glbizzia.com (Shang-Tong Li)


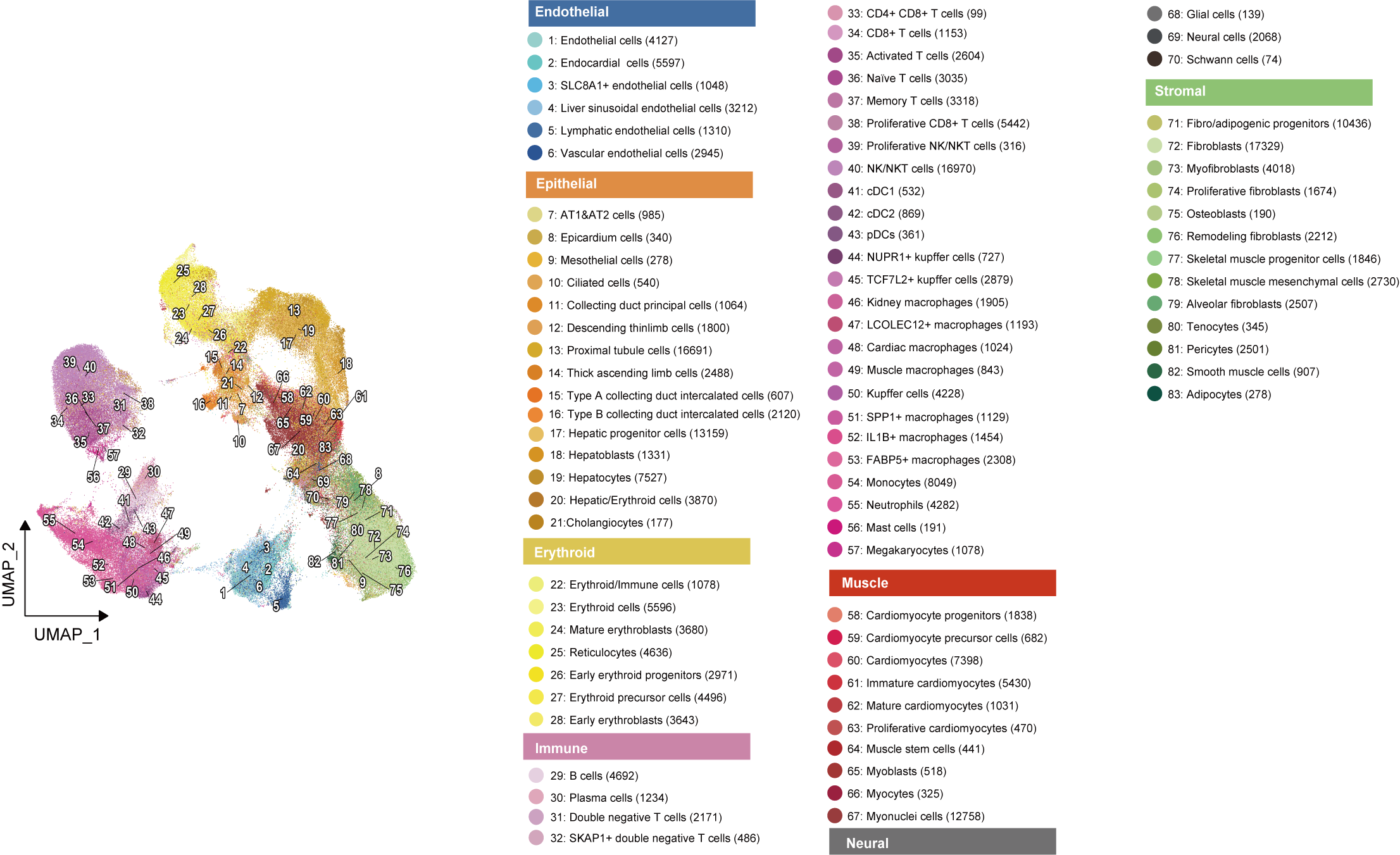


**Figure S1 UMAP visualization colored by seven annotated cell lineages.** Cell types and counts per lineage are listed.


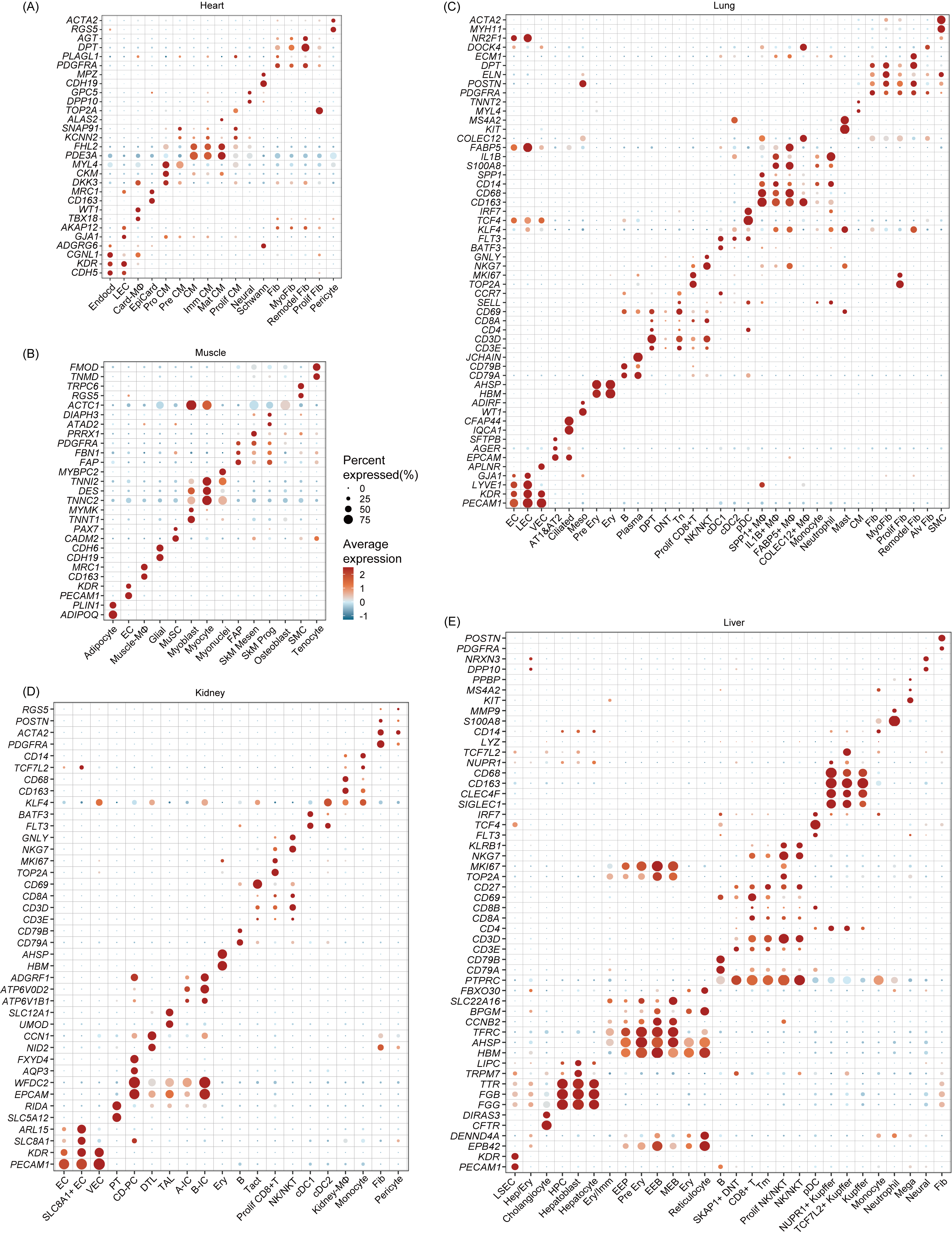


**Figure S2 Dot plot showing specific markers for cell types in the heart (A), muscle (B), lung (C), kidney (D), and liver (E).** The x-axis represents different cell types and the y-axis represents marker genes. Dot size indicates the fraction of cells expressing each gene, while color intensity reflects the mean expression level. The abbreviations of cell types are as follows: Endothelial cells (EC), Endocardial cells (Endocard), SLC8A1+ endothelial cells (SLC8A1+ EC), Liver sinusoidal endothelial cells (LSEC), Lymphatic endothelial cells (LEC), Vascular endothelial cells (VEC), AT1&AT2 cells (AT1&AT2), Epicardium cells (EpiCard), Mesothelial cells (Meso), Ciliated cells (Ciliated), Collecting duct principal cells (CD-PC), Descending thin limb cells (DTL), Proximal tubule cells (PT), Thick ascending limb cells (TAL), Type A collecting duct intercalated cells (A-IC), Type B collecting duct intercalated cells (B-IC), Hepatic progenitor cells (HPC), Hepatoblasts (Hepatoblast), Hepatocytes (Hepatocyte), Hepatic/Erythroid cells (Hep/Ery), Cholangiocytes (Cholangiocyte), Erythroid/Immune cells (Ery/Imm), Erythroid cells (Ery), Mature erythroblasts (MEB), Reticulocytes (Reticulocyte), Early erythroid progenitors (EEP), Erythroid precursor cells (Pre Ery), Early erythroblasts (EEB), B cells (B), Plasma cells (Plasma), Double negative T cells (DNT), SKAP1+ double negative T cells (SKAP1+ DNT), CD4+ CD8+ T cells (DPT), CD8+ T cells (CD8+ T), Activated T cells (Tact), Naïve T cells (Tn), Memory T cells (Tm), Proliferative CD8+ T cells (Prolif CD8+T), Proliferative NK/NKT cells (Prolif NK/NKT), NK/NKT cells (NK/NKT), cDC1 (cDC1), cDC2 (cDC2), pDCs (pDC), NUPR1+ kupffer cells (NUPR1+ Kupffer), TCF7L2+ kupffer cells (TCF7L2+ Kupffer), Kidney macrophages (Kidney-MΦ), COLEC12+ macrophages (COLEC12+ MΦ), Cardiac macrophages (Card-MΦ), Muscle macrophages (Muscle-MΦ), Kupffer cells (Kupffer), SPP1+ macrophages (SPP1+ MΦ), IL1B+ macrophages (IL1B+ MΦ), FABP5+ macrophages (FABP5+ MΦ), Monocytes (Monocyte), Neutrophils (Neutrophil), Mast cells (Mast), Megakaryocytes (Mega), Cardiomyocyte progenitors (Pro CM), Cardiomyocyte precursor cells (Pre CM), Cardiomyocytes (CM), Immature cardiomyocytes (Imm CM), Mature cardiomyocytes (Mat CM), Proliferative cardiomyocytes (Prolif CM), Muscle stem cells (MuSC), Myoblasts (Myoblast), Myocytes (Myocyte), Myonuclei cells (Myonuclei), Glial cells (Glial), Neural cells (Neural), Schwann cells (Schwann), Fibro/adipogenic progenitors (FAP), Fibroblasts (Fib), Myofibroblasts (MyoFib), Proliferative fibroblasts (Prolif Fib), Osteoblasts (Osteoblast), Remodeling fibroblasts (Remodel Fib), Skeletal muscle mesenchymal cells (SkM Mesen), Skeletal muscle progenitor cells (SkM Prog), Alveolar fibroblasts (Alv Fib), Tenocytes (Tenocyte), Pericytes (Pericyte), Smooth muscle cells (SMC), Adipocytes (Adipocyte).


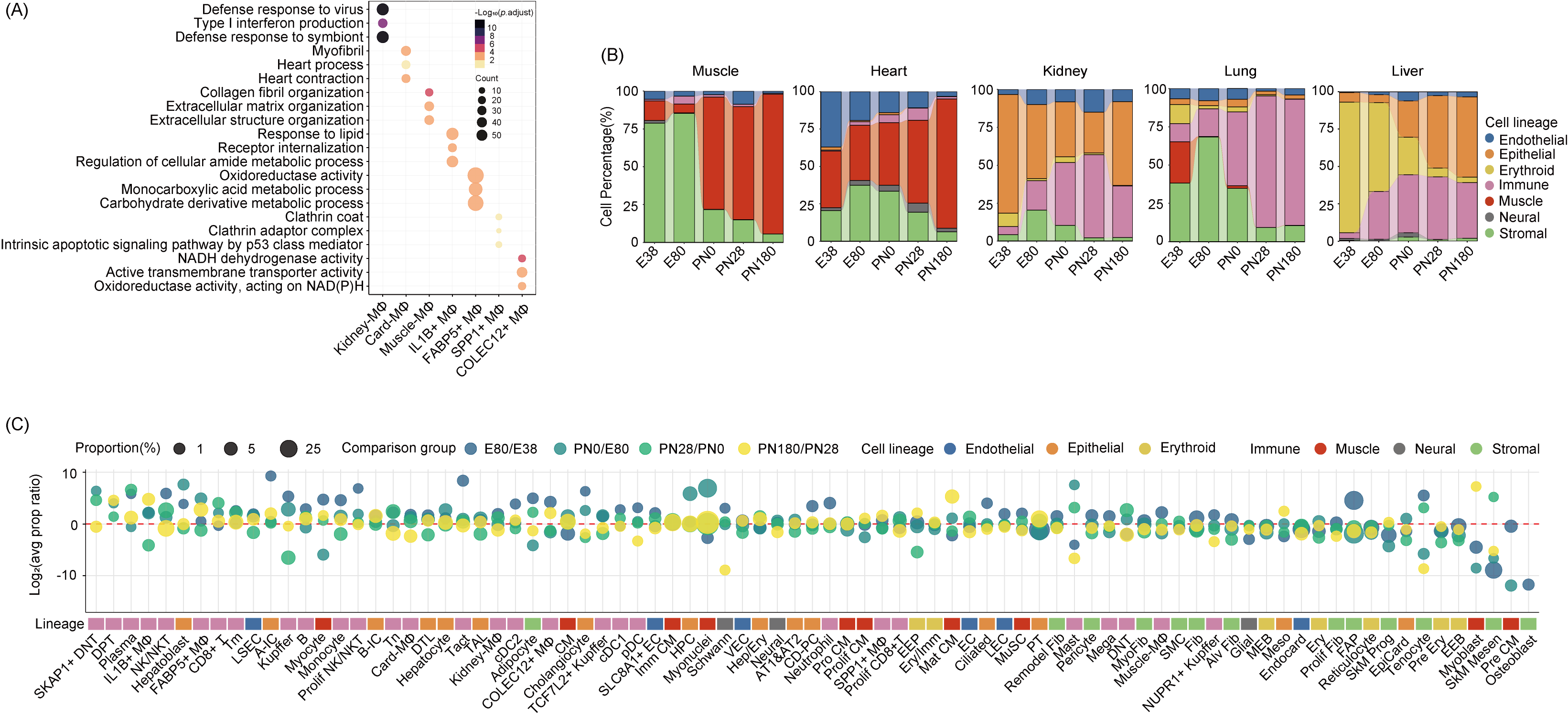


**Figure S3 GO enrichment analysis of macrophage subtypes and developmental dynamics of cell type composition.** (A) Gene Ontology (GO) enrichment analysis of the differentially expressed genes for each macrophage subtype, with dot size representing the number of genes involved and color indicating the statistical significance (-Log_10_(*p*.adjust)). (B) Bar plot showing the dynamics of cell lineage proportions during development in five tissues. (C) Dot plot displaying the dynamic ratios of cell types across development stages. The x-axis represents cell types, and the y-axis shows the ratios of each cell type between adjacent developmental stages. Dot size corresponds to the magnitude of the ratio. Cell abbreviations are the same as in Figure S2.


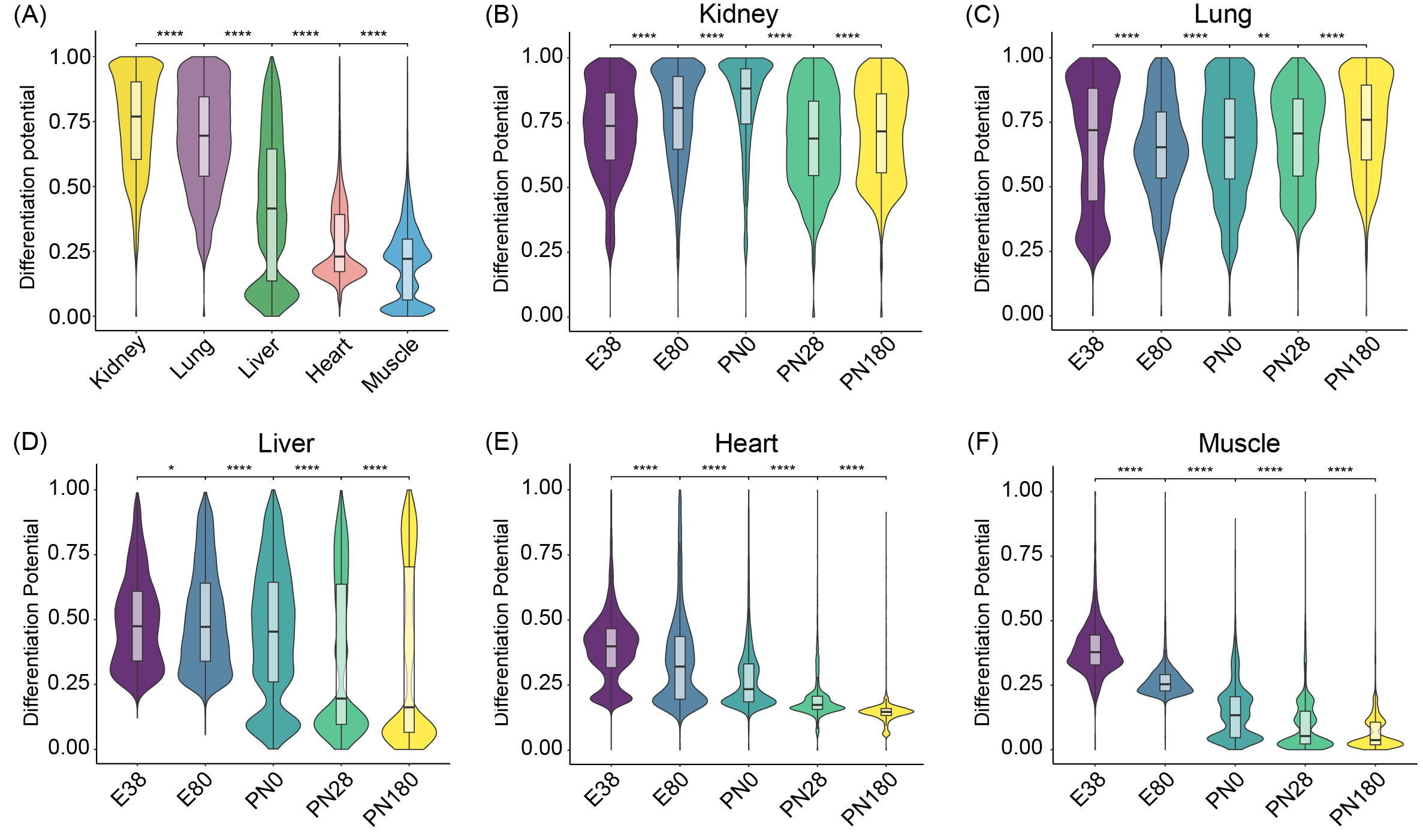


**Figure S4 Dynamics of differentiation potential across tissues and development.** (A-F) Stemness scores were calculated using CytoTRACE for multiple tissues (A) and for individual tissue including kidney (B), lung (C), liver (D), heart (E), and muscle (F) at different developmental stages. The significance of differences in stemness scores between adjacent developmental stages, assessed using an unpaired Wilcoxon rank-sum test (two-sided) (ns, not significant, *p* > 0.05; **p* ≤ 0.05; ***p* ≤ 0.01; ****p* ≤ 0.001; *****p* ≤ 0.0001).


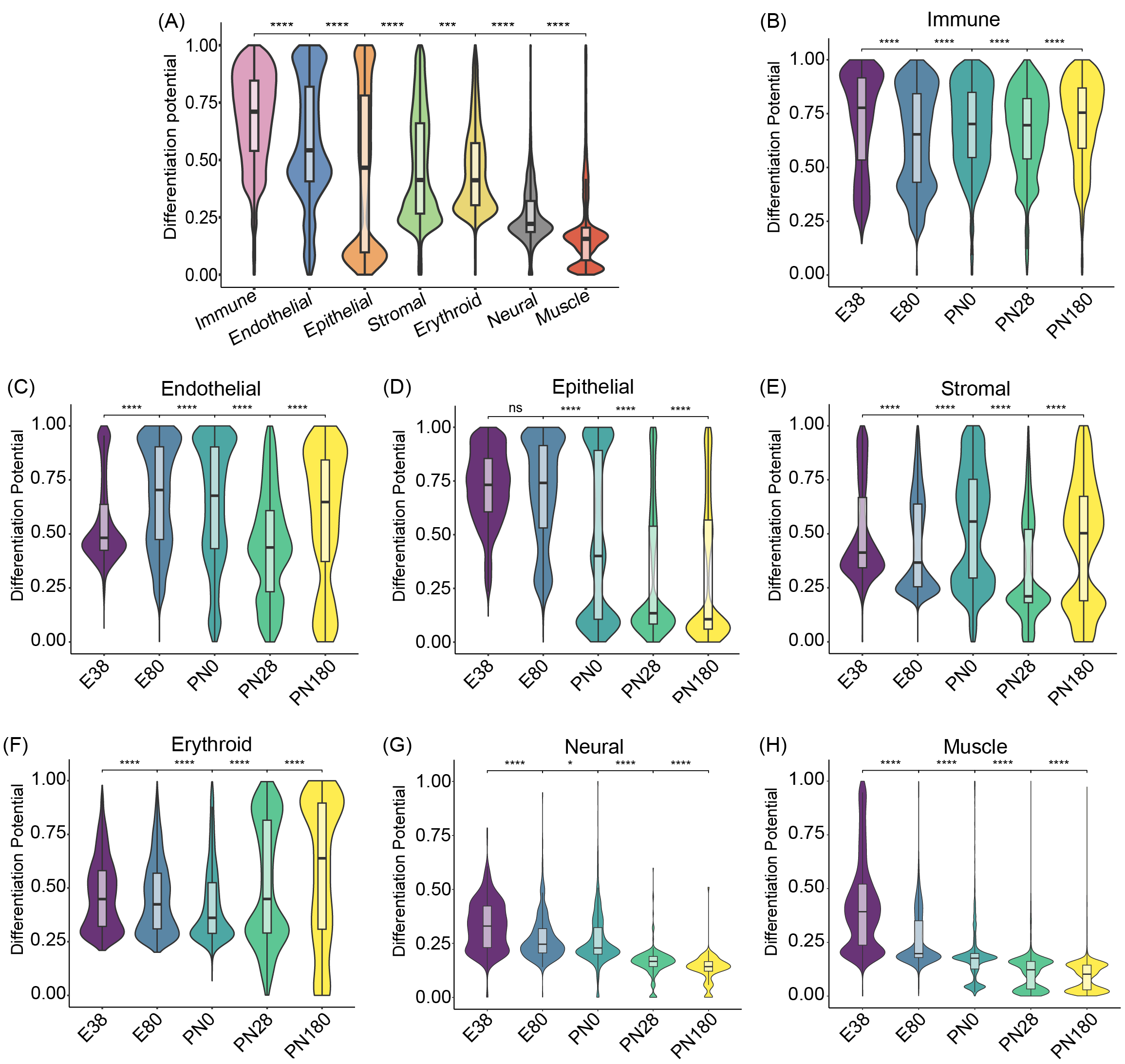


**Figure S5 Lineage-Specific Dynamics of Differentiation Potential across Development.** (A-H) Stemness scores were calculated using CytoTRACE for major cell lineages (A), Immune (B), Endothelial (C), Epithelial (D), Stromal (E), Erythroid (F), Neural (G), and Muscle (H) in different development stages. The significance of differences in stemness scores between adjacent developmental stages, assessed using an unpaired Wilcoxon rank-sum test (two-sided) (ns, not significant, *p* > 0.05; **p* ≤ 0.05; ***p* ≤ 0.01; ****p* ≤ 0.001; *****p* ≤ 0.0001).


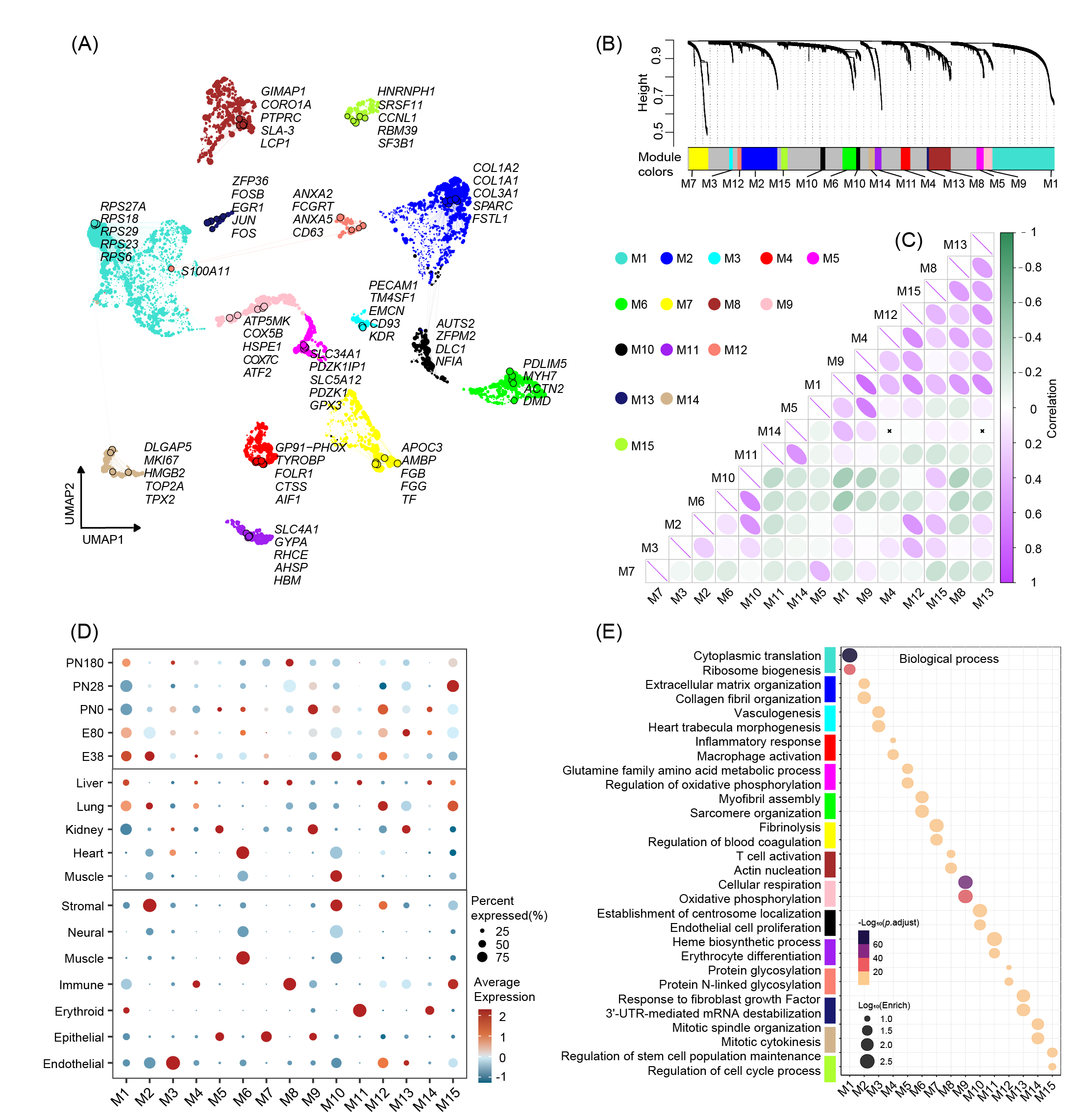


**Figure S6 Spatiotemporally coordinated gene co-expression modules reveal lineage- and tissue-specific regulatory programs during pig development.** (A) UMAP visualization of the high-dimensional weighted gene co-expression network (hdWGCNA), where each node represents a single gene and edges denote co-expression relationships. Nodes are colored by module assignment, and point size is scaled by module membership (kME). The top four to five hub genes per module are labeled, highlighting key regulators within each co-expression cluster. (B) Hierarchical clustering dendrogram showing the relationships of the 15 co-expression modules. (C) Correlation analysis among module eigengenes demonstrating inter-module relationships and identifying higher-order functional groupings of co-expression modules associated with major developmental lineages. (D) Module eigengene expression across lineages, tissues, and developmental stages. Dot size represents the proportion of cells expressing genes within the module, and color intensity indicates average expression level.

(E) Gene Ontology (GO) enrichment analysis for each co-expression module, showing representative biological processes associated with module-specific functional themes.


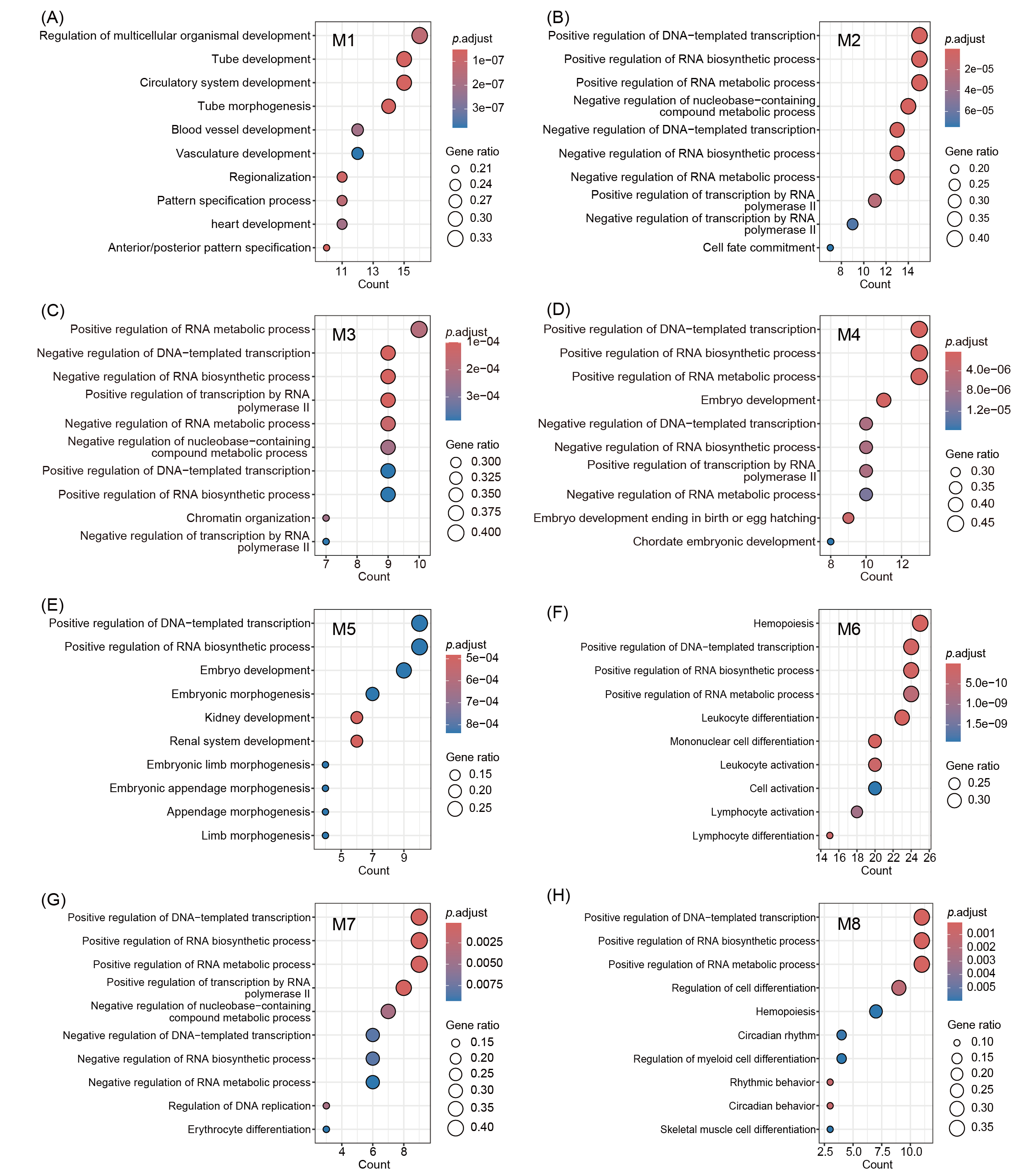


**Figure S7 Enrichment of Gene Ontology (GO) Biological Processes in transcription factor (TF) co-expression modules.** The dot plot shows selected significantly enriched GO terms for each module, with dot size representing the number of genes involved and color indicating the statistical significance (*p*.adjust).

**
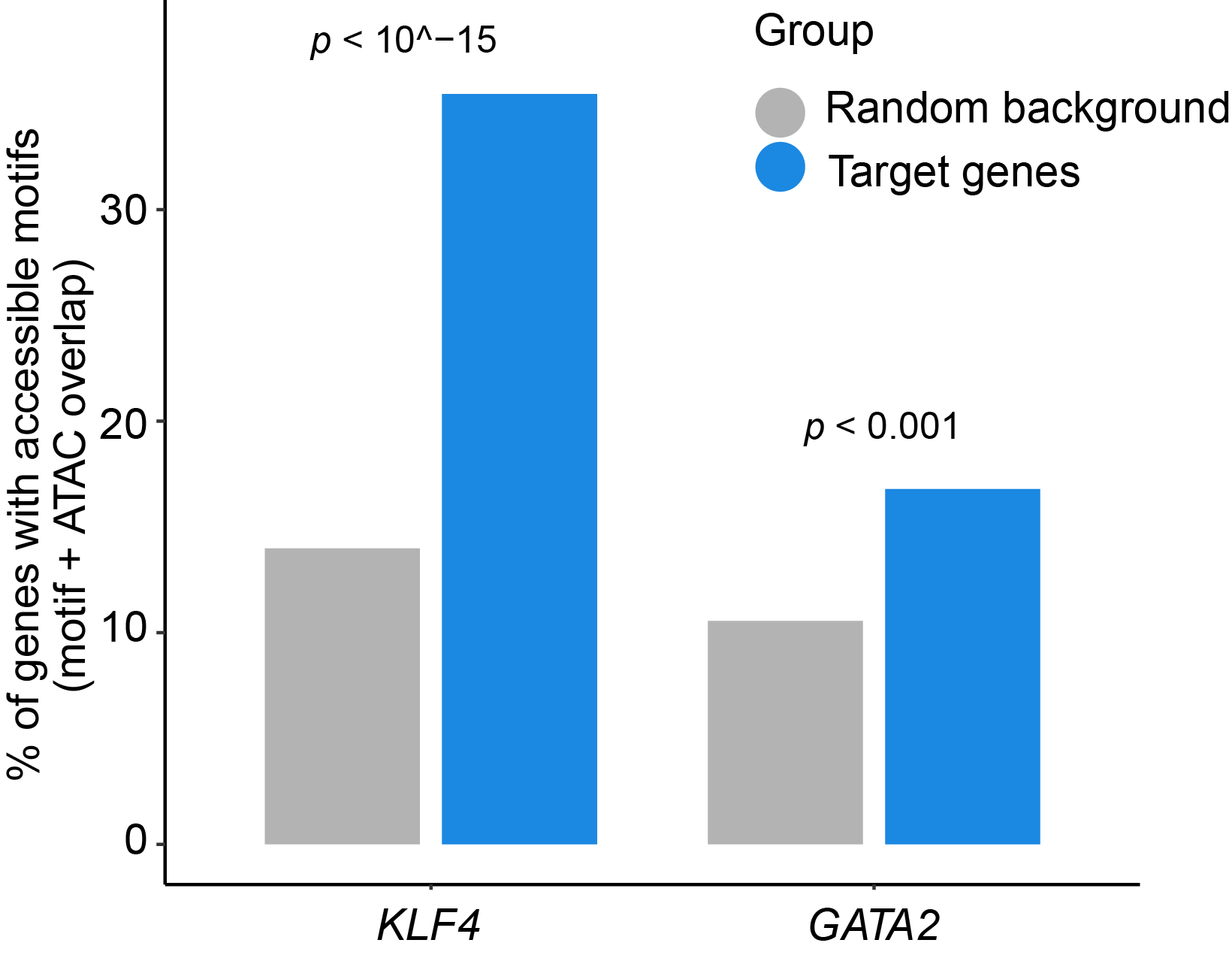
**

**Figure S8 Chromatin accessibility enrichment analysis for predicted *KLF4* and *GATA2* regulon targets within the M8 immune-lineage module.** Chromatin accessibility of predicted transcription factor binding sites was evaluated using published porcine immune-cell snATAC-seq data. For each transcription factor, promoter regions of predicted target genes identified by SCENIC were assessed for overlap between TF binding motifs and accessible chromatin peaks and compared with a random background gene set (n = 1000).


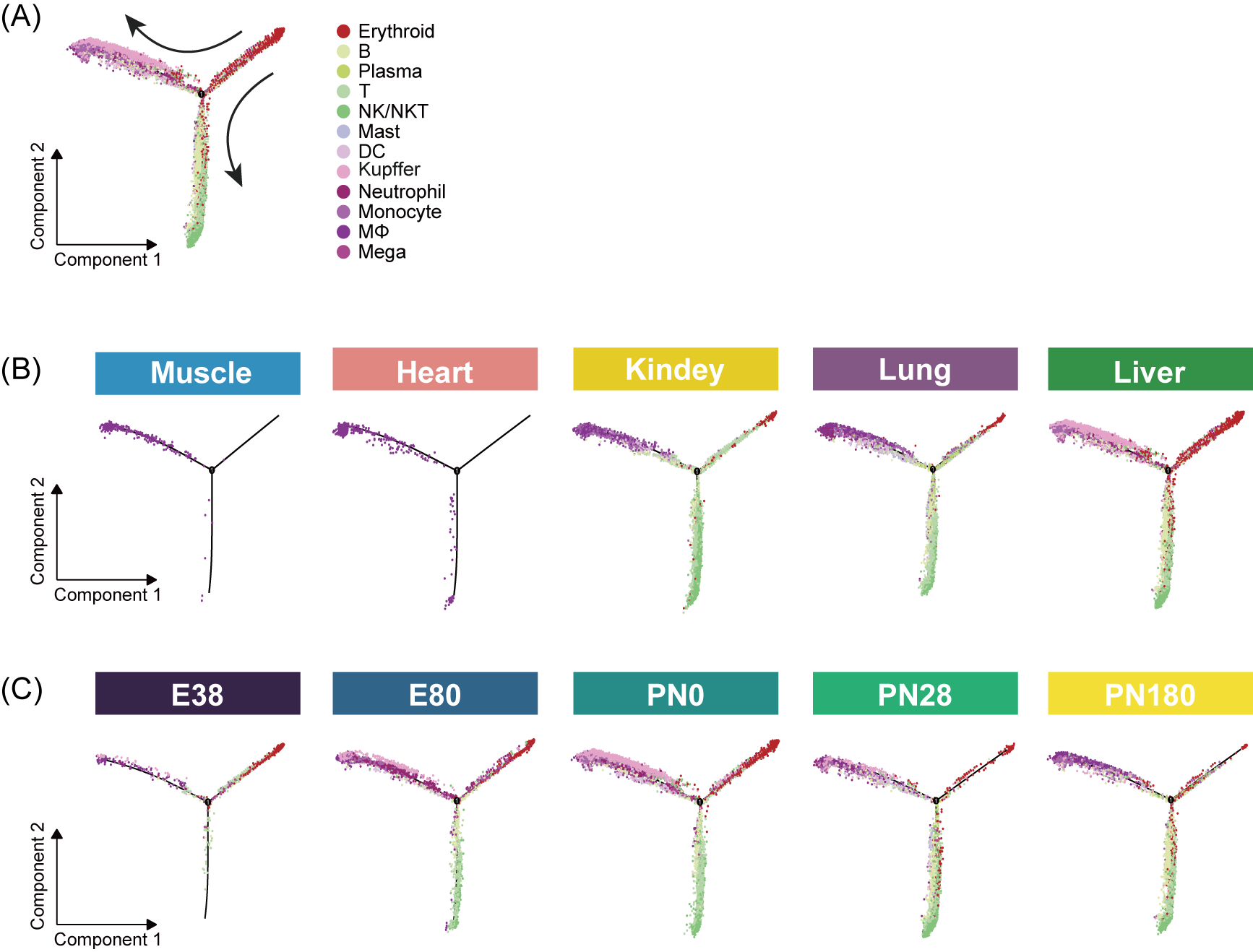


**Figure S9 Composition of the immune cell trajectory across tissues and developmental stages.** (A) Immune cells development trajectory colored by cell types. (B and C) The distribution of cells within the trajectory is shown colored by cell types identity across different tissues (B) and developmental stages (C).


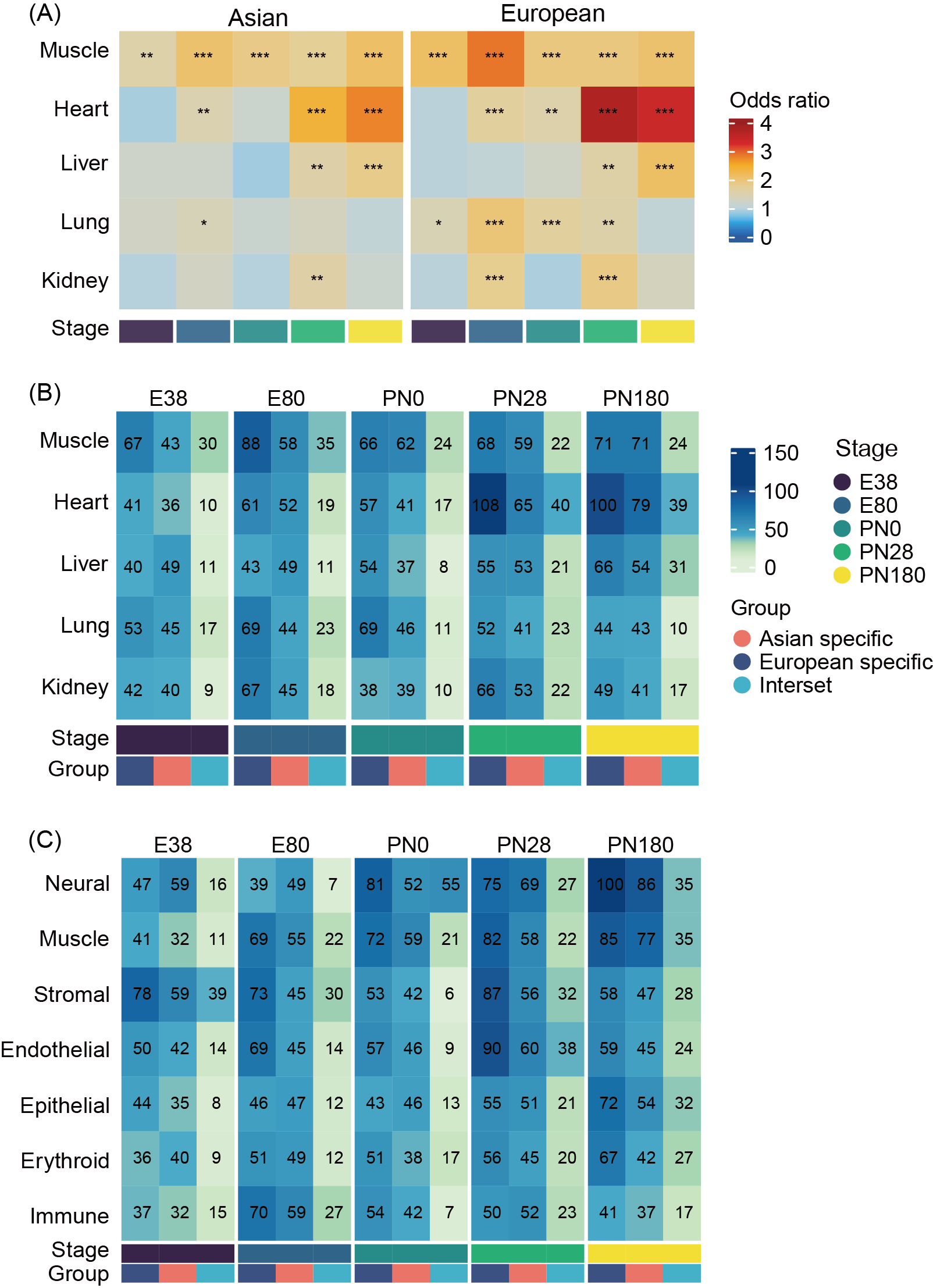


**Figure S10 Analysis of quantitative specificity and selection in gene datasets from Eurasian pigs.** (A) The heatmaps show Fisher’s exact test odds ratios (ORs) quantifying the overlap between tissue-specific gene sets and selective gene sets identified in Eurasian domestic pigs. Heatmap colors represent enrichment ORs. Statistical significance was assessed using Fisher’s exact test followed by Benjamini–Hochberg FDR correction across all tested lineage–selection comparisons. Asterisks indicate FDR-adjusted significance levels (**q* < 0.05, ***q* < 0.01, ****q* < 0.001). (B) Heatmap displaying the number of overlapping genes between tissue-specific gene datasets and selective gene datasets in Eurasian pigs. Color intensity corresponds to the count of genes under selection. (C) Heatmap displaying the number of overlapping genes between cell lineage-specific gene datasets and selective gene datasets in Eurasian pigs. Color intensity scales represent the count of selected overlapping genes.


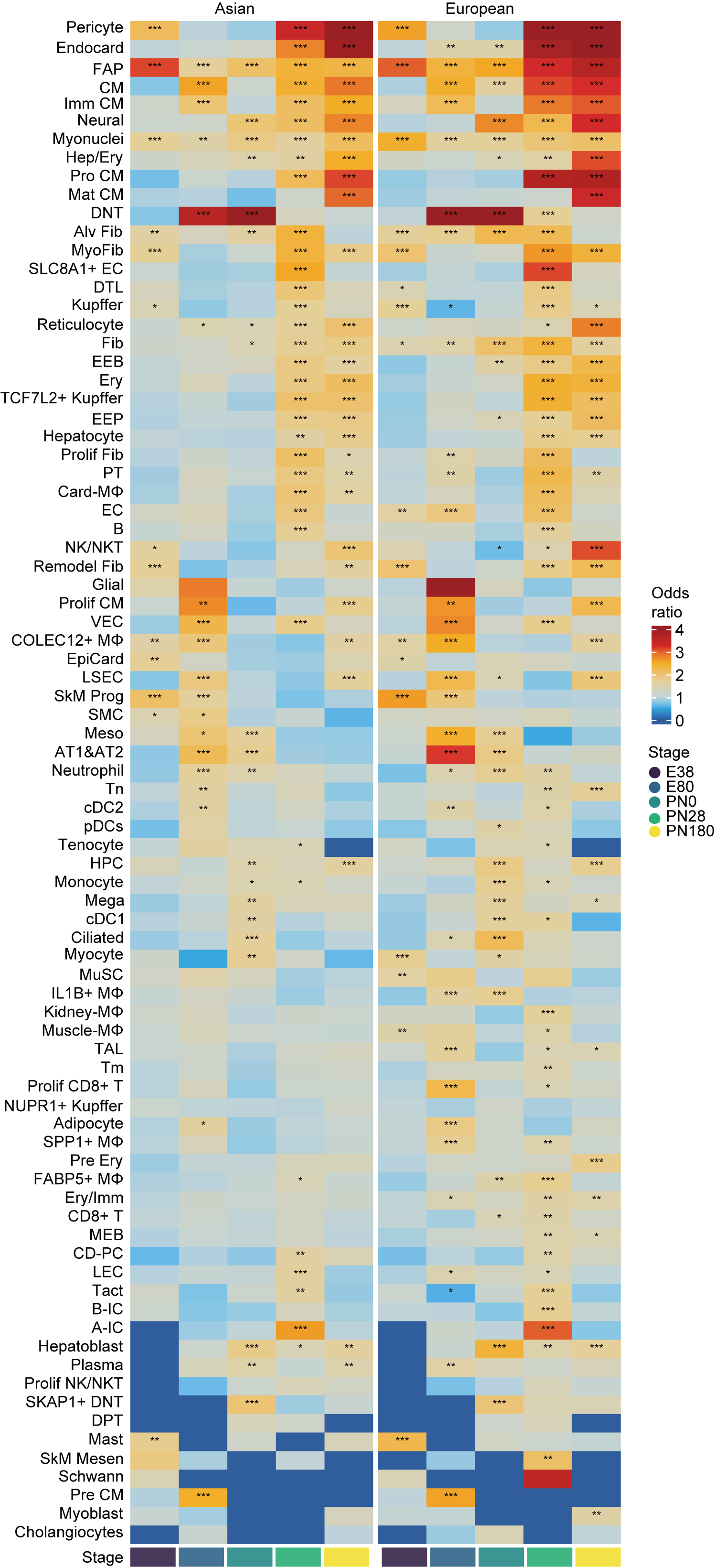


**Figure S11 Association between cell type-specific gene sets and selective signatures in Eurasian pigs.** The heatmaps show Fisher’s exact test odds ratios (ORs) quantifying the overlap between cell type-specific gene sets and selective gene sets identified in Eurasian domestic pigs. Heatmap colors represent enrichment ORs. Statistical significance was assessed using Fisher’s exact test followed by Benjamini–Hochberg FDR correction across all tested lineage–selection comparisons. Asterisks indicate FDR-adjusted significance levels (**q* < 0.05, ***q* < 0.01, ****q* < 0.001).


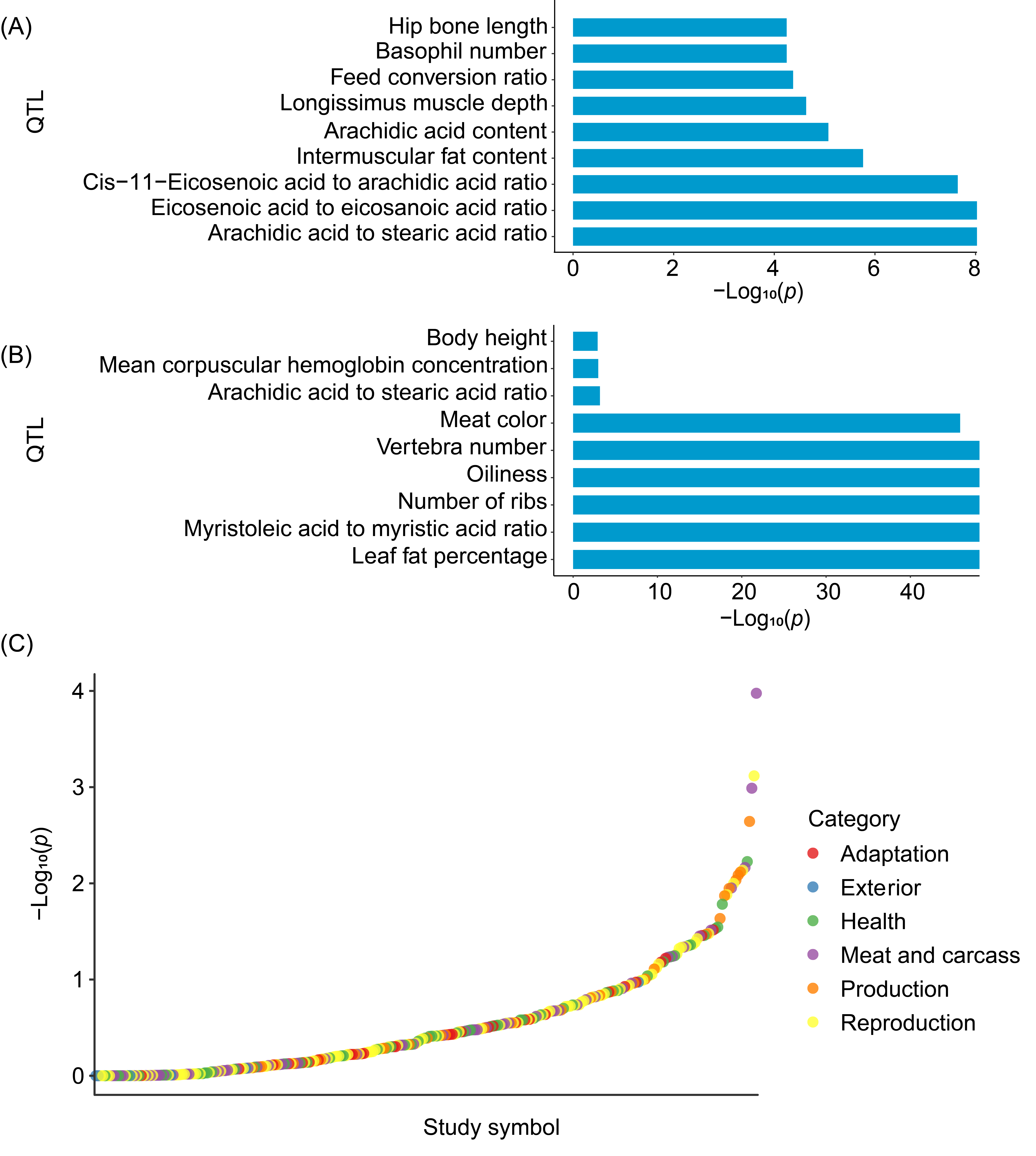


**Figure S12 QTL enrichment analysis of domestication regions in Asian and European pig breeds and association analysis of the *MYOT* gene.** (A) Significantly enriched QTL terms for the domestic selective regions of Asian pigs. Enrichment significance is based on adjusted *p* obtained from GALLO (multiple-testing correction). (B) Significantly enriched QTL terms for the domestic selective regions of European pigs. Enrichment significance is based on adjusted *p* obtained from GALLO (multiple-testing correction). (C) Point plot shows the association between *MYOT* and multiple complex traits based on gene-based association analysis (S-PrediXcan/S-MultiXcan). Each point represents the Bonferroni-corrected *p* for the association of *MYOT* with a given trait. The full names of the traits and their corresponding corrected p-values are listed in Table S12.

**
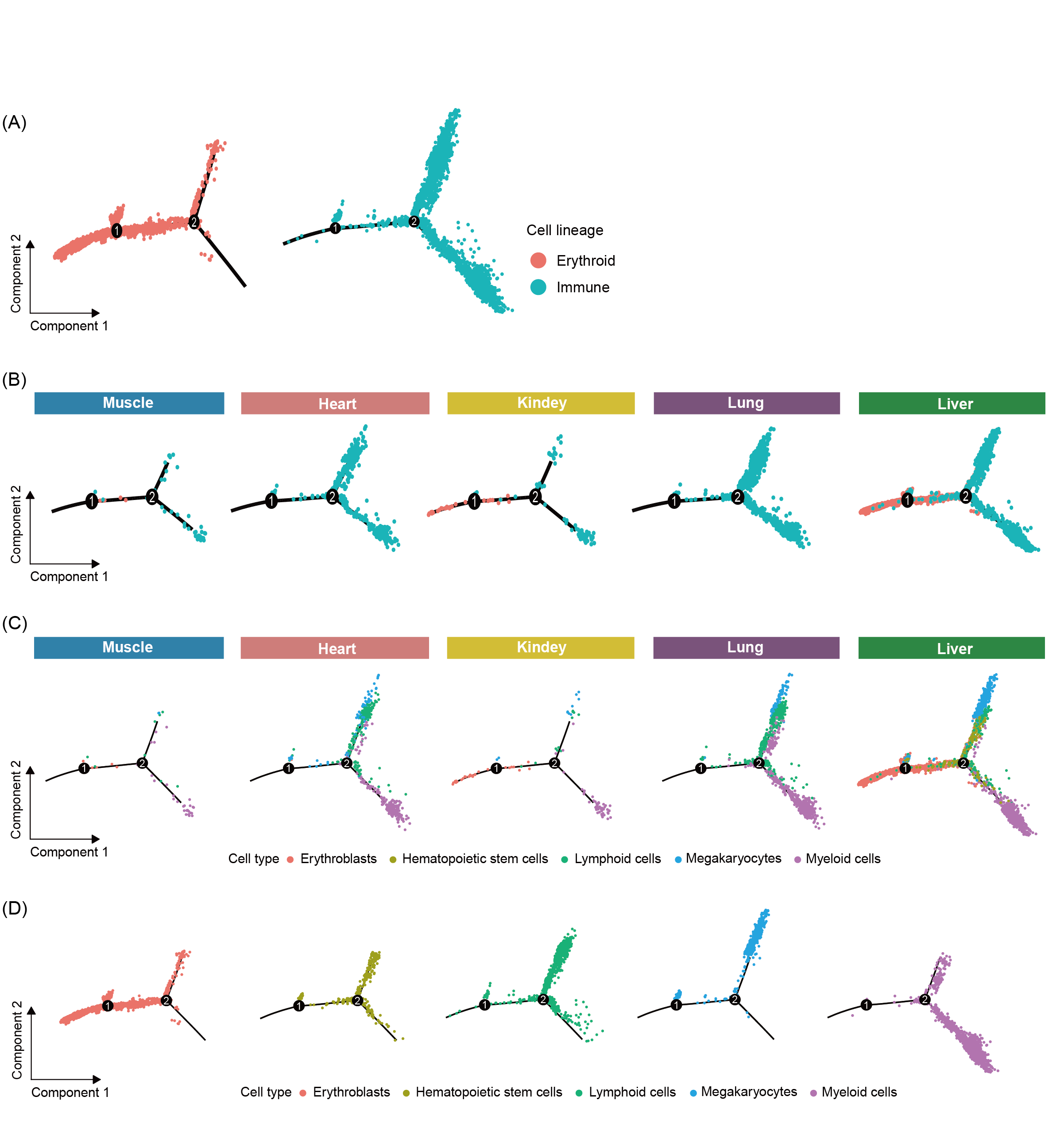
**

**Figure S13 Composition of the human immune cells and erythroid cells trajectory across tissues.** (A) Immune cells and erythroid cells development trajectory colored by cell lineages. (B) The distribution of cells within the trajectory is shown colored by lineages identity across different tissues. (C) The distribution of cells within the trajectory is shown colored by cell types identity across different tissues. (D) Immune cells and erythroid cells development trajectory colored by cell types.


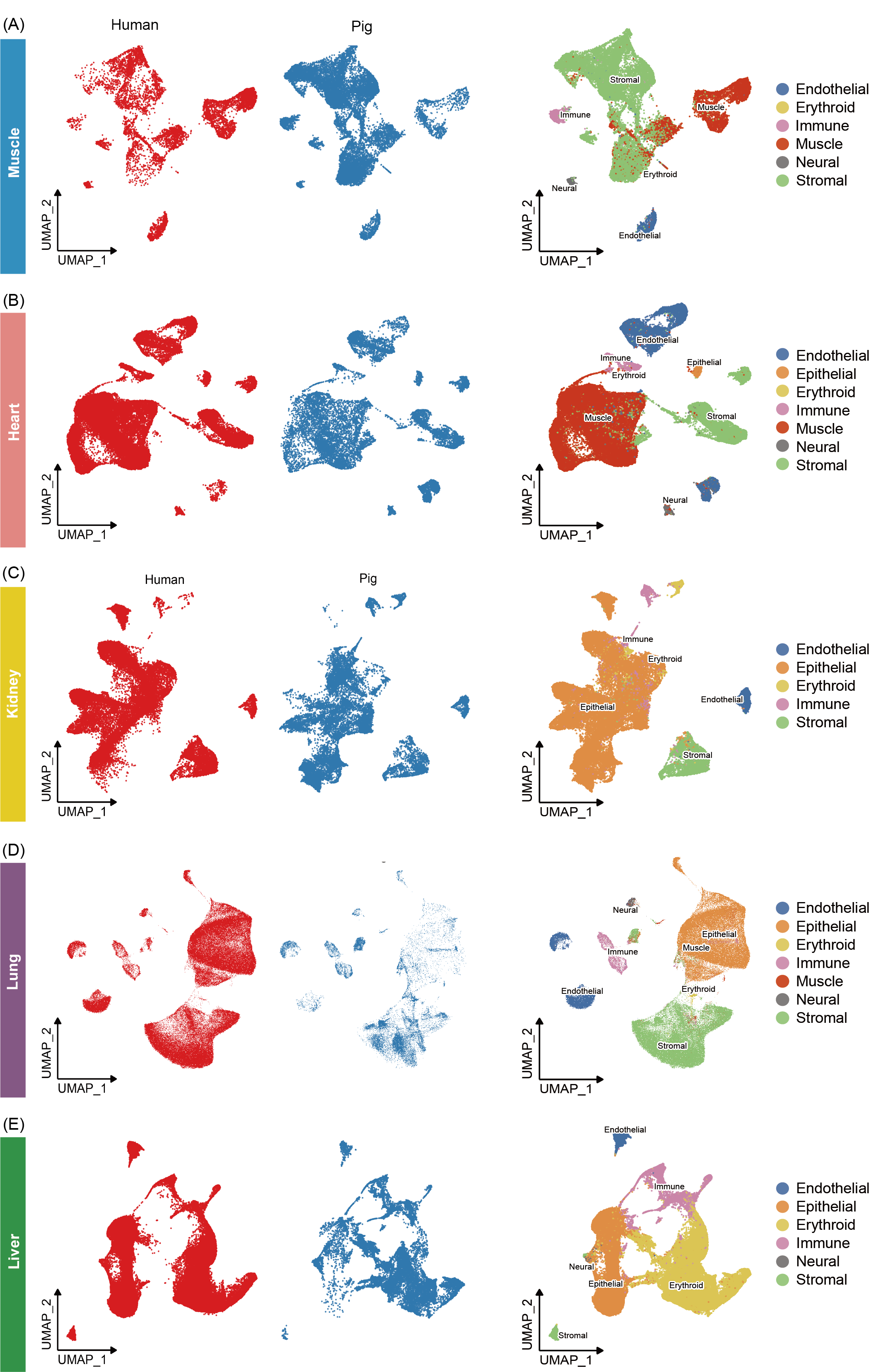


**Figure S14 Cross-species UMAP visualization of five tissues.** (A) UMAPs of the muscle tissue, colored by species (Human, Pig) and cell lineage. (B) UMAPs of the heart tissue, colored by species (Human, Pig) and cell lineage. (C) UMAPs of the kidney tissue, colored by species (Human, Pig) and cell lineage. (D) UMAPs of the lung tissue, colored by species (Human, Pig) and cell lineage. (E) UMAPs of the liver tissue, colored by species (Human, Pig) and cell lineage.


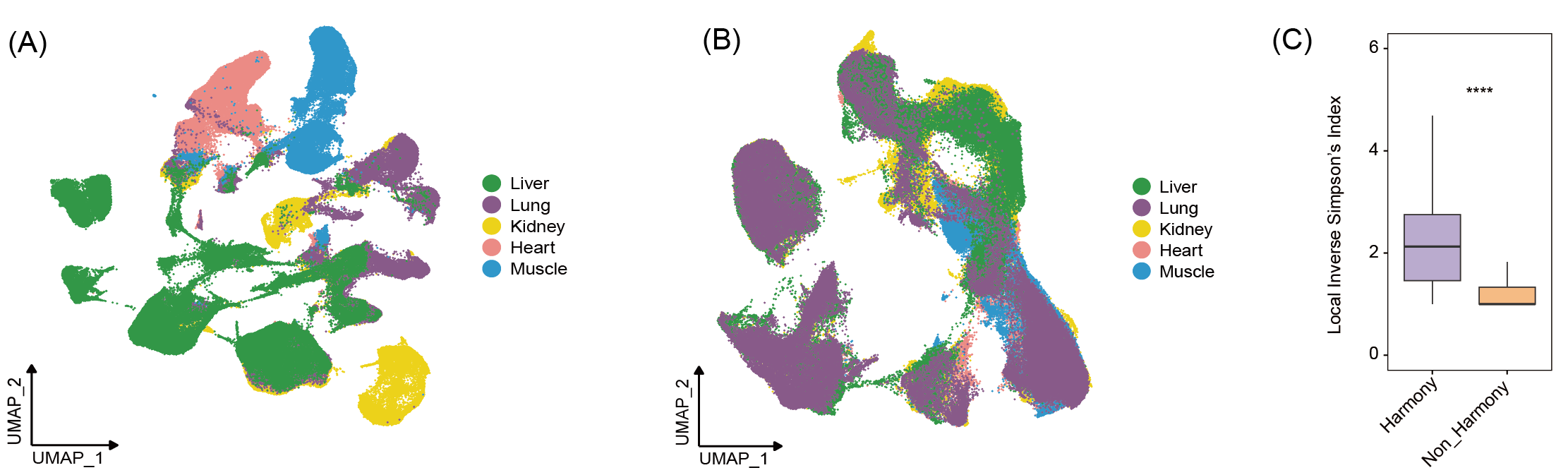


**Figure S15 Integration and evaluation of multi-tissue single-cell data with the Harmony algorithm.** (A) UMAP visualization of five pig tissues before Harmony integration. (B) UMAP visualization of five pig tissues after Harmony integration. (C) Local Inverse Simpson’s Index indicate assess both batch mixing and the preservation of biological heterogeneity.
